# Sleep disturbance as transdiagnostic mediator between adverse childhood experiences and psychopathology in children and adolescents: A structural equation modeling meta‐analysis

**DOI:** 10.1002/jcv2.12156

**Published:** 2023-03-23

**Authors:** Jianlin Liu, Wen Lin Teh, Rachel Hsiao Shen Tan, Yoke Boon Tan, Charmaine Tang, Nisha Chandwani, Mythily Subramaniam

**Affiliations:** ^1^ Research Division Institute of Mental Health Singapore Singapore; ^2^ Department of Psychosis Institute of Mental Health Singapore Singapore; ^3^ Department of Mood & Anxiety Institute of Mental Health Singapore Singapore

**Keywords:** adverse childhood experiences, children, meta‐analysis, psychopathology, sleep disturbance

## Abstract

**Background:**

Increasing research efforts have focused on understanding why some individuals develop severe psychopathology after exposure to adverse childhood experiences (ACEs). Sleep disturbances (insomnia, nightmares, and sleep disorders) are prevalent sequelae of ACEs and associated with psychopathology; however, there is no meta‐analytic evidence on whether sleep disturbance functions as a transdiagnostic mediator in the relationship between ACEs and psychopathology (internalizing/externalizing disorders and psychosis) in children and adolescents.

**Methods:**

Systematic searches in three databases (PubMed; PsycINFO; Web of Science) identified 98 articles (*N* = 402,718; age range 1–17 years) and the present study used a novel two‐stage meta‐analytic structural equation model to investigate whether ACEs predict psychopathology through sleep disturbance. Subgroup analyses determined potential biases due to study design (cross‐sectional vs. longitudinal) and geographical differences (Western vs. non‐Western countries). Sensitivity analyses evaluated the influence of early childhood (<5 years old) and overlapping symptoms (i.e., nightmares and trauma symptoms) on model stability.

**Results:**

The pooled correlations among ACEs, sleep disturbance, and psychopathology were significant; the effect sizes ranged from moderate to high (*r* = 0.21 to *r* = 0.29). The indirect effect from ACEs via sleep disturbance to psychopathology was significant (*β* = 0.05, 95% CI [0.04, 0.06]). The direct effect of ACEs on psychopathology was significant (*β* = 0.18, 95% CI 0.13–0.24). Subgroup analyses revealed larger effects for cross‐sectional studies than longitudinal studies (Δ χ^2^ (3) = 9.71, *p* = 0.021). Sensitivity analyses revealed stable and consistent results.

**Conclusions:**

The present meta‐analytic results indicate that sleep disturbance is a transdiagnostic mediator in the relationship between ACEs and psychopathology among children and adolescents. Further research is required to determine the synergistic effects between sleep disturbance and other risk mechanisms, and elucidate the complex pathways that lead to disorder in the aftermath of childhood adversities.

**Key points**

- Sleep disturbance is prevalent in children and adolescents with ACEs and psychopathology
- Sleep disturbance is shown to be a transdiagnostic mediator in the positive relationship between ACEs and psychopathology in children and adolescents
- Results support the need for comprehensive assessment of sleep disturbance in ACEs‐exposed individuals regardless of their diagnosis
- Results support the implementation of existing psychological treatment for sleep disturbance as a unified transdiagnostic ACEs intervention

## INTRODUCTION

Adverse childhood experiences (ACEs) are stressful and traumatic events that occur from birth till 18 years of age and result in harm, potential harm, or threat of harm to a child (Leeb et al., 2007). ACEs include child maltreatment (i.e., all forms of abuse and neglect), domestic violence, loss of a parent (e.g. sudden death or divorce), living in dysfunctional home environments (e.g. parents who are struggling with mental illness), bullying, accidents, war, terrorism, and natural disasters (Gibson et al., 2016; Sideli et al., 2020).

Accumulating research evidence shows that individuals exposed to ACEs have an elevated risk of developing mental disorders and ACEs are associated with more severe psychopathology (i.e., ranging from symptomatology to diagnosis of mental disorders) (Gibson et al., 2016; Hoppen & Chalder, 2018; Sideli et al., 2020). Neurobiological models explain that ACEs can severely dysregulate the stress response system which is highly sensitive during its development in childhood (Elbers et al., 2017). Consequently, the inability to effectively regulate stress and negative emotions increases the risk of experiencing early symptoms of mental disorders in childhood and adolescence, as well as the development of mental disorders later in life (Laskemoen et al., 2021; Miu et al., 2022).

ACEs are also reported to disrupt the circadian system through sleep disturbance (i.e., insomnia, hypersomnia, nightmares, frequent awakenings, shorter duration of sleep, and sleep disorders) (Laskemoen et al., 2021; Schønning et al., 2022). Both stress and sleep are closely related and circadian system dysfunction may further dysregulate the stress response system after exposure to ACEs. A vicious cycle forms when chronic activation of the stress response system directly impairs brain areas regulating sleep or indirectly increases the release of hormones that stimulate heightened attention and vigilance, which in turn cause immediate and persistent sleep disturbance (Laskemoen et al., 2021; Schønning et al., 2022). Ultimately, both stress dysregulation and disturbed sleep create a dysfunctional internal system that is unable to effectively maintain important functions for health restoration; prolonged functional impairment increases the risk of developing psychopathology (Elbers et al., 2017; Laskemoen et al., 2021).

The extant literature reports that ACEs are associated with sleep disturbance (a path), and sleep disturbance is associated with psychopathology (b path) (Laskemoen et al., 2021; Schønning et al., 2022); hence, sleep disturbance is a potential mediator linking ACEs to psychopathology. One previous meta‐analysis (*k* = 17; cross‐sectional and longitudinal studies) determined the association between ACEs and sleep disturbance (a path) in children and adolescents between ages 5–18 years (Schønning et al., 2022). ACEs were defined as child maltreatment and witnessing interpersonal violence, while sleep disturbance was focused on symptoms of insomnia, nightmares, and shorter sleep duration. Overall, ACEs were associated with a three‐fold increased risk in experiencing symptoms of insomnia and nightmares, and shorter duration of sleep compared to those who were not exposed to ACEs (Schønning et al., 2022). Further research is required to address the limitations in this meta‐analysis: (a) include a broader range of ACEs (e.g. natural disasters and accidents) and sleep disturbance (e.g. hypersomnia and sleep disorders), (b) test the moderating effects of study designs (i.e., cross‐sectional vs. longitudinal) and geographical differences (i.e., West vs. non‐Western countries) as culture may influence the prevalence and severity of ACEs (Schønning et al., 2022); and (c) evaluate the association between sleep disturbance and psychopathology (b path).

Another meta‐analysis (*k* = 25; longitudinal studies) examined the association between sleep disturbance and first onset of major mental disorders (b path) in early adulthood; it should be noted, however, that this meta‐analysis did not consider ACEs and included studies comprised both youth and adult samples up to age 30 years (Scott et al., 2021). Sleep disturbance was defined as insomnia symptoms, insomnia and fatigue, hypersomnia, and sleep disorders. Major mental disorders were defined as depressive, bipolar, and psychotic disorders. Overall, sleep disturbance in youth was longitudinally associated with increased risk of major mental disorders in early adulthood (Scott et al., 2021). Further research is required to address the limitations in this meta‐analysis: (a) examine a broader range of sleep disturbance (e.g. nightmares) and mental disorders (e.g. PTSD), (b) investigate the full spectrum of psychopathology (e.g. subsyndromal mental conditions), and (c) evaluate the association between sleep disturbance and psychopathology (b path) in children and adolescents only. In terms of assessment measures, both previous meta‐analyses reported that sleep disturbance was commonly measured by self‐report or observer‐report, and few studies relied on objective measures (e.g. actigraphy). Notably, preliminary evidence suggests that these measures are comparable in measuring sleep disturbance and the type of measure did not significantly bias the overall results (Schønning et al., 2022; Scott et al., 2021).

Taking the findings and limitations from these two previous meta‐analyses together, there is a lack of meta‐analytic evidence on the association between sleep disturbance and psychopathology in children and adolescents (b path), and both meta‐analyses did not examine the association between ACEs and psychopathology in children and adolescents (c path). Hence, a new meta‐analysis is warranted to investigate the mediating role of sleep disturbance in the pathway between ACEs and psychopathology in children and adolescents. Moreover, ACEs‐related psychopathology in childhood and adolescence could share pertinent risk mechanisms with ACEs‐related psychopathology in adulthood; thus, more research is required to elucidate risk mechanisms (as early intervention targets) in the relationship between ACEs and psychopathology in children and adolescents, and progress in this area is critical for increasing the specificity and efficacy of ACEs interventions (Lorenc et al., 2020). Given that ACEs are associated with a broad spectrum of psychopathology and overlapping comorbid conditions (i.e., multifinality), current research is focused on elucidating transdiagnostic factors (i.e., common processes across psychopathology) such as sleep disturbance that may be targeted in treatment (Dalgleish et al., 2020; Laskemoen et al., 2019).

Recent advances in evidence synthesis methodology have enabled researchers to conduct meta‐analytic structural equation modeling (MASEM) to investigate hypothesized mediation models (Cheung, 2015a; Jak, 2015). This involves a two‐stage approach: (a) creating a pooled correlation matrix; and (b) fitting a mediation model (i.e., ACEs → sleep disturbance → psychopathology) to the data (Cheung, 2015a). Meta‐analytic structural equation modeling has several strengths: (a) ability to account for sample size differences across the elements of the pooled correlation matrix; (b) ability to account for the nested nature of correlations; and (c) the use of full information maximum likelihood estimation to handle missing data (Cheung, 2015a).

In contrast to conducting a single mediation study which is constrained by its sample size, MASEM offers greater statistical power as correlations and covariances are pooled across multiple studies (Cheung, 2015a; Jak, 2015). Therefore, MASEM is appropriate to test the statistical significance and magnitude of the indirect effect of ACEs on psychopathology via sleep disturbance in children and adolescents. In terms of clinical utility, the new information gleaned from this meta‐analysis will inform clinicians in their assessment and intervention strategies for ACEs and sleep disturbance.

The objective of this study was to perform MASEM to test the mediating role of sleep disturbance in the pathway between ACEs and psychopathology in children and adolescents. In view of current evidence, we focused on the transdiagnostic approach; psychopathology was broadly defined as internalizing symptoms (i.e., mood and anxiety disorders), externalizing symptoms (i.e., disruptive behavior disorders and attention deficit‐hyperactivity disorder) and psychotic‐like experiences (e.g. hallucinations and delusions), as well as subsyndromal and full diagnosis of mental disorders in children and adolescents (Polanczyk et al., 2015). Overall, the hypothesized mediation model included both the indirect (i.e., ACEs to psychopathology via sleep disturbance) and direct pathways between ACEs and psychopathology. We hypothesized that sleep disturbance would mediate the positive relationship between ACEs and psychopathology.

## METHODS

The present study was conducted in accordance with the Preferred Reporting in Systematic Reviews and Meta‐Analysis Guidelines and the study protocol was registered in the National Institute for Health Research's International prospective register of systematic review PROSPERO on 20 May 2022 (registration number CRD42022333272).

### Search strategy

We used a combination of MeSH‐terms and keywords to identify all relevant studies on the concept of ACEs, sleep disturbance, and psychopathology in children and adolescents (see Table S1 for full search strategy). A summarized version of the search for each concept: (child abuse or maltreat* or ACE*) and (sleep* or insomnia or parasomnia or hypersomnia or nightmare or night terror) and (child* or teen* or adolescen*). A systematic literature search was conducted in MEDLINE (PubMed), PsycINFO, and Web of Science from 1 January 1994 to 23 May 2022. We included this search limit to coincide with the introduction of the Diagnostic and Statistical Manual of Mental Disorders, 4^th^ Edition (DSM‐IV); thus, our results are relevant to current clinical practice. Hand searches were conducted for three key trauma journals (Child Abuse & Neglect, Journal of Traumatic Stress, and Psychological Trauma: Theory, Research, Practice, and Policy).

### Inclusion and exclusion criteria

Studies were eligible for inclusion in this meta‐analysis if they met the following criteria:

1. The study was peer‐reviewed and published full‐text in English scientific journals.
2. The study reported empirical and quantitative data.
3. The study's sample comprised participants below the age of 18 years (≥80%); a study with only a subsample of participants below the age cut‐off could be included if specific data for the eligible subgroup were available to allow effect sizes to be estimated independently.
4. The study specified an assessment of ACEs (i.e., broadly defined as any stressful and traumatic events that occurred while growing up or before the age of 18) using self‐report, other‐report or official records.
5. The study specified an assessment procedure for and nature of any sleep disturbance using self‐report, other‐report or official records.

Exclusion criteria:

1. Studies were systematic reviews, meta‐analyses, case reports, dissertations, conference abstracts or book chapters.
2. Studies that did not report an association between ACEs and sleep disturbance (e.g. reported on ACEs only or sleep disturbance only).
3. Studies that focused on adults.
4. Studies that were unable to provide correlational data after contacting the corresponding authors.

### Selection of studies

All studies were independently evaluated by at least two authors to determine study inclusion. First, three authors (WLT, RHST, and YBT) screened each article by their title and abstract. Next, two authors (JL and WLT) screened all potential articles by full text. Any discrepancies were resolved through consensus discussions. All references were handled in Endnote X9 reference manager program.

### Data extraction

Data from each study were independently extracted by three authors (JL, RHST, and YBT) and disagreements were resolved through consensus discussions. Meta‐analytic structural equation modeling requires correlation effect sizes to be coded, pooled, and estimated in the structural equation model (Cheung, 2015a); thus, we primarily extracted correlations coefficients of relevant associations among ACEs, sleep disturbance, and psychopathology from each study. However, not all studies reported correlation coefficients; we extracted and converted the next available effect size to correlation coefficients based on formulas for converting among effect sizes (Borenstein et al., 2011). For studies that reported linear regressions, we extracted standardized regression coefficients and estimated correlation coefficients (Peterson & Brown, 2005). For path analyses, we extracted path coefficients from within path models and used the implied function from the metaSEM package in R to convert path coefficients to correlation coefficients. All effect sizes were coded such that positive values indicated more severe sleep disturbance or psychopathology. The original authors were contacted whenever there were insufficient data to determine correlation effect sizes.

We addressed the unit‐of‐analysis problem (i.e., violation of independent effect sizes) by aggregating multiple effect sizes within a study to form a composite effect size. For example, we used the Fisher's Z‐transformation and then back transformed the Fisher's Z coefficients to correlation coefficients (Borenstein et al., 2011). If a study reported both suicidality and depression, we prioritized and extracted the effect size for depression only as depression is a mental disorder while suicidality is a symptom of depression. For longitudinal studies, we focused on extracting the effect size for the association between the predictor (e.g. ACEs) and the final wave outcome (e.g. psychopathology) before age 18. If two or more studies reported findings from the same dataset, we selected the study that reported the most effect sizes and relevant associations. In addition to correlation effect sizes, the following study characteristics were extracted: sample size, participants' age, sample sex distribution, study design (i.e., cross‐sectional or longitudinal), types of ACEs, sleep disturbance and psychopathology, and type of instruments used to measure ACEs, sleep disturbance, and psychopathology.

### Meta‐analysis

Two‐stage MASEM was performed in R (version 4.1.3) using the metaSEM package (Cheung, 2015b). In Stage 1, the random effects model was used to obtain a pooled correlation matrix among all coded variables. Full information maximum likelihood estimation was used to pool correlation matrices. Effect sizes were interpreted as small (*r* = 0.10), moderate (*r* = 0.20), and high (*r* = 0.30) (Gignac & Szodorai, 2016). To evaluate the degree of homogeneity of effect sizes, the total heterogeneity of weighted mean effects (Cochran's Q) and the total variation across studies attributable to heterogeneity (*I* ^*2*^) were calculated. *I* ^*2*^ values above 75% indicate considerable heterogeneity. Heterogeneity in each correlation set (i.e., ACEs – sleep disturbance; sleep disturbance – psychopathology; ACEs – psychopathology) were examined based on *τ* ^*2*^ coefficients.

In Stage 2, weighted least squares estimation were used to fit the hypothesized mediation model to the resultant pooled correlation matrix from Stage 1. Given that all paths would be estimated (i.e., saturated model where fit indices will always indicate perfect fit to the data), mediation was tested by evaluating the significance of the direct and indirect effects based on 95% confidence intervals (Jak, 2015). The parameter estimate was considered significant if the 95% confidence interval around the estimated parameter did not include zero.

### Sensitivity analyses

Nightmares may be assessed as both sleep disturbance and symptom of PTSD (Schønning et al., 2022); thus, the first sensitivity analysis excluded studies which assessed for nightmares (as sleep disturbance) and did not remove the nightmare symptom item from their PTSD outcome measure (*k* = 6). Parasomnias are more prevalent in early childhood (1–5 years) than middle childhood/adolescence (Schønning et al., 2022); hence, the second sensitivity analyses further excluded studies with children below the age of 5 years (*k* = 6).

### Subgroup analyses

Study design (i.e., cross‐sectional vs. longitudinal research) is a potential moderator as cross‐sectional studies are more likely to report inflated mediation estimates than longitudinal studies (O'Laughlin et al., 2018). Geographic difference (i.e., Western vs. non‐Western countries) is another potential moderator as some ACEs (e.g. emotional abuse and neglect) are considered culturally acceptable practices in Asian communities and may be underreported (Kumari, 2020); hence, studies from non‐Western countries may have smaller mediation effects than studies from Western countries. Both study design and geographic difference were selected for subgroup analyses as each study could be clearly coded and divided into fairly equal subgroups with no overlaps. Further, cultural and geographic differences of ACEs are significant research gaps to be addressed (Schønning et al., 2022). On the other hand, ACEs exposure (i.e., single vs. multiple) was not considered as some ACEs overlap (e.g. physical and emotional abuse) and tend to co‐occur, which create difficulties in determining the frequency and severity of exposure (Neuner, 2023).

Subgroup analyses determined (a) if all paths and indirect effect were replicated in each subgroup; and (b) whether subgroups significantly differed from each other (Jak, 2015; Jak & Cheung, 2018). First, studies were split into two groups based on study design (cross‐sectional = 0; longitudinal = 1) and geographical difference (non‐Western countries = 0; Western countries = 1) where Western countries included Americas, Europe, and Australia. Next, we tested the equivalence of the direct effects across groups by constraining the effects to be equal across subgroups (i.e., increasing the degrees of freedom). A moderating effect is present when the χ^2^ statistic increases significantly (*p* < 0.05), which indicates that the subgroups significantly differed from each other (Jak, 2015; Jak & Cheung, 2018).

### Study quality

The methodological quality of studies was independently assessed by three authors (JL, RT, and YB) using the National Institutes of Health Quality Assessment Tool for Observational Cohort and Cross‐Sectional Studies (available at http://nhlbi.nih.gov). This tool has 14 criteria which evaluates the methodological rigor of study designs. Each study was evaluated according to the 14 criteria and given an overall rating of “good” (≥11), “fair” (7–10), or “poor” (≤6). Ratings were compared and differences resolved by consensus.

## RESULTS

### Selection and inclusion of studies

Our initial search identified 1793 articles. After removal of duplicates (*k* = 450), 1343 articles were screened by title and abstract and 1023 articles were excluded. The remaining 320 articles were screened by full‐text for eligibility. 222 articles were excluded based on the exclusion criteria and 98 articles (*N* = 402,718) were included in this meta‐analysis. Figure S1 depicts the flow chart of the study selection process.

Table S2 presents the study characteristics of the included studies: country, study design, type of sample, sample size, sample age, and assessment measure and type of ACEs, sleep disturbance, and psychopathology. The majority of the studies were based on cross‐sectional designs (*k* = 74; 75.5%) and conducted in Western countries (*k* = 68; 69.4%). The assessment measures for sleep disturbance varied across studies and the more frequently used measures included The Pittsburgh Sleep Quality Index, The Insomnia Severity Index, and The Child Behavior Checklist. Internalizing disorders (depression, anxiety, and PTSD) were the most commonly studied psychopathology and were assessed by a range of self‐ and observer‐reports.

### Meta‐analytic structural equation modeling Stage 1: Pooled correlations

We used a random effects Stage 1 model to pool the correlation matrices and fit the data. The pooled correlations among ACEs, sleep disturbance, and psychopathology were significant; the effect sizes ranged from moderate to high (*r* = 0.21 to *r* = 0.29). The Q statistic was significant (Q_(135)_ = 6278.45, *p* < 0.001) and the average *I* ^*2*^ of the three correlation coefficients was about 0.98; thus, there were considerable between study heterogeneity (i.e., a large part of the variance is at study level). Table S3 presents the pooled correlations and between study heterogeneity coefficients.

### Meta‐analytic structural equation modeling Stage 2: Mediation model

We fitted the hypothesized mediation model to the pooled Stage 1 correlation matrices in which ACEs directly predicted psychopathology and indirectly predicted psychopathology through sleep disturbance. We estimated a saturated model (i.e., a model with zero degrees of freedom) where all variables were associated with each other and model fit could not be assessed.

The paths from ACEs to sleep disturbance (*β* = 0.21, 95% CI 0.18–0.25), and sleep disturbance to psychopathology (*β* = 0.25, 95% CI 0.20–0.30) were significant; forest plots are presented in Figure S2 (path a) and Figure S3 (path b). The indirect effect from ACEs via sleep disturbance to psychopathology was significant (*β* = 0.05, 95% CI [0.04, 0.06]). Furthermore, there was a significant direct effect of ACEs on psychopathology (*β* = 0.18, 95% CI 0.13–0.24); forest plot is presented in Figure S4 (path c). Table 1 presents all parameter estimates and 95% CIs.

**TABLE 1:** Parameter estimates and 95% confidence intervals based on Stage 2 random‐effect analyses.

	Estimate	LLCI	ULCI
^*β*^ACEs → Sleep disturbance	0.21	0.18	0.25
^*β*^Sleep disturbance → Psychopathology	0.25	0.20	0.30
^*β*^ACEs → Psychopathology	0.18	0.13	0.24
Indirect effect	0.05	0.04	0.06

Abbreviations: ACEs, Adverse Childhood Experiences; LLCI, lower level confidence interval; ULCI, upper level confidence interval.

### Sensitivity analyses

The overall sensitivity analyses revealed stable results where the significant indirect effect from ACEs via sleep disturbance to psychopathology was replicated (*β* = 0.05, 95% CI [0.04, 0.07]) when studies which assessed for nightmares and did not remove the nightmare symptom item from their PTSD outcome measure (*k* = 6), as well as studies which included children below the age of 5 years (*k* = 6) were excluded from the analysis.

### Subgroup Stage 1: Random‐effects analyses

We first pooled and estimated different correlation matrices in the group of studies with cross‐sectional designs and the group of studies with longitudinal designs (Table S4). The proportions of between studies variance (*I* ^*2*^) within the subgroup for longitudinal studies were smaller than they were in the total sample, indicating that study design explained part of the between‐study heterogeneity. We next pooled and estimated different correlation matrices in the group of studies with Western samples and the group of studies with non‐Western samples (Table S5). The proportions of between studies variance (*I* ^*2*^) within the subgroups were comparable to the total sample; hence, indicating that geographical differences do not account for the between‐study heterogeneity.

### Subgroup Stage 2: Testing moderation of effects by study design and geographical differences

The hypothesized mediation model was replicated in both subgroups for study design. All direct and indirect effects were replicated in both cross‐sectional and longitudinal studies, but there was a significant difference between models (Δ χ^2^ (3) = 9.71, *p* = 0.021). The path from ACEs to psychopathology was significantly smaller for longitudinal studies (*β* = 0.10, 95% CI 0.04–0.17) than cross‐sectional studies (*β* = 0.21, 95% CI 0.15–0.27); the path from ACEs to sleep disturbance was also significantly smaller for longitudinal studies (*β* = 0.17, 95% CI 0.12–0.22) than cross‐sectional studies (*β* = 0.22, 95% CI 0.18–0.27) (Table S6). The hypothesized mediation model was replicated in both subgroups for geographical differences (Table S7). All direct and indirect effects were replicated in both Western and non‐Western studies; however, the difference between models was not significant (Δ χ^2^ (3) = 1.95, *p* = 0.582).

### Quality assessment

The median quality rating was 7 (out of 14); 9 articles were graded as good quality; 54 as fair and 35 as poor. Only half of the studies reported clear inclusion/exclusion criteria (*k* = 51; 52%), while very few studies reported their power calculation (*k* = 8; 8.2%) and none had outcome assessors blinded to the exposure status of participants. Moreover, most studies were based on cross‐sectional designs; thus, only longitudinal studies were able to meet most of the criteria. Table S8 summarizes the quality assessment.

## DISCUSSION

This is the first meta‐analysis to investigate and provide empirical evidence on the mediating role of sleep disturbance in the pathway between ACEs and psychopathology in children and adolescents. This study addressed several important research gaps in the literature. First, the present study employed novel MASEM approach to link current meta‐analytic evidence on the positive association between ACEs and sleep disturbance in children and adolescents (Schønning et al., 2022), as well as the positive association between sleep disturbance and mental disorders in early adulthood (Scott et al., 2021). Our results indicate that sleep disturbance is a partial mediator in the relationship between ACEs and psychopathology. Hence, sleep disturbance is a risk factor and one pathway through which ACEs contribute to the onset and maintenance of psychopathology. Moreover, our results are aligned with the extant literature where multiple mechanisms are proposed to link the positive association between ACEs and psychopathology. For example, a recent meta‐analysis reported that emotion dysregulation was a partial mediator in the positive association between ACEs and psychopathology (Miu et al., 2022). Given that emotion dysregulation and circadian dysfunction are related and may co‐occur (Elbers et al., 2017), both emotion dysregulation and sleep disturbance could potentially interact and exacerbate the severity of psychopathology.

Second, the present study was focused on children and adolescents to address the current gap in knowledge on the associations among ACEs, sleep disturbance, and psychopathology in this population. Our results support the hypothesized mediation model, which suggest that the onset of ACEs‐related psychopathology may occur earlier during childhood and it is not limited to early adulthood. This highlights the immediate and enduring impact of ACEs on mental health. Further, the most prevalent psychopathology among included studies in this meta‐analysis was mood and anxiety disorders; thus, the development of these ACEs‐related psychopathology in childhood and adolescence may be the prodrome phase for schizophrenia and related psychoses (Scott et al., 2021). This view is aligned with growing evidence on the mediating role of affective disorders in the positive relationship between ACEs and psychosis (Isvoranu et al., 2017; Liu et al., 2020).

Third, the present study investigated a broad range of ACEs, sleep disturbance, and psychopathology dimensions and tested a transdiagnostic mediation model (i.e., analyzed all three key constructs as a whole). Overall, our results support the transdiagnostic mediation model where ACEs (beyond child maltreatment) predict psychopathology (from symptoms to disorder) via sleep disturbance. Notably, the transdiagnostic perspective accounts for the co‐occurrence of multiple ACEs and sleep disturbance, as well as the high prevalence of psychiatric comorbidity in ACEs‐exposed populations.

Finally, the present study used subgroup analyses to address methodological constraints associated with conducting individual mediation studies. Our results revealed that the hypothesized mediation model was replicated in both subgroups for cross‐sectional and longitudinal studies. This finding provides strong empirical support on the causal role of sleep disturbance in the positive association between ACEs and psychopathology in children and adolescents. However, the effects were larger in cross‐sectional studies as compared to longitudinal studies. This is consistent with previous observations of smaller effect sizes in longitudinal studies (Scott et al., 2021).

Subgroup analyses were also conducted to address the potential effects of culture on the prevalence and severity of ACEs. Previous meta‐analysis on child maltreatment and sleep in children and adolescents included a predominant Western sample and the results may not be generalizable to non‐Western populations (Schønning et al., 2022). Some have suggested that cultural variations on the definitions of emotional abuse and emotional neglect may account for underreporting on these types of ACEs as compared to physical abuse, sexual abuse, and physical neglect (Kumari, 2020). Accordingly, the present study tested the moderating effect of geographical differences (i.e., Western vs. non‐Western countries) and did not observe any significant difference. Therefore, the present study provides evidence on the consistent negative impact of ACEs on sleep disturbance and psychopathology across countries and communities, which supports the notion that ACEs as a whole is a global public health concern.

### Clinical implications

The present study has important implications for clinical practice. Our results highlight that sleep disturbance is a prevalent sequela of ACEs and clinicians should conduct a comprehensive assessment of sleep disturbance in ACEs‐exposed children and adolescents regardless of their diagnosis. This includes assessment for co‐occurrence of multiple types of sleep disturbance. For example, nightmares could cause frequent sleep disruptions and lead to insomnia (Laskemoen et al., 2021). Moreover, nightmares are common symptoms of trauma and post‐traumatic stress, hence, those who report persistent nightmares should be further assessed for PTSD (Laskemoen et al., 2021).

In terms of intervention, the present study provided compelling evidence on sleep disturbance as a transdiagnostic mediator and the corresponding moderate to large effect sizes underscore the importance of treating sleep disturbance to improve psychopathology in children/adolescents exposed to ACEs. Recent clinical trials have shown that cognitive behavioral therapy (CBT) for sleep disturbance is efficacious for adults suffering from depression (Cunningham & Shapiro, 2018), PTSD (Ho et al., 2016), and psychosis (Freeman et al., 2017). There is also growing evidence on the efficacy of third‐wave CBT for sleep disturbance (Rash et al., 2019). Our results point to the importance of implementing these therapies as a unified transdiagnostic ACEs intervention, and to determine if the therapeutic effects may extend to children and adolescents as previous intervention studies have predominantly focused on adults.

### Limitations

The present study has some limitations that must be considered. First, about a third (*k* = 35; 35.7%) of the included studies were rated as poor quality and this could have introduced some bias to our results. Poor quality was attributed in part to unreported power calculations; hence, this must be taken into account as the effect sizes from some individual studies may not be sufficiently powered to test for mediation. Moreover, most studies were based on cross‐sectional designs and would not have met the full quality criteria. Second, we were not able to fully account for the high heterogeneity in this study; one possible source could be the heterogenous study measures of sleep disturbance. The decision to combine heterogenous studies into one meta‐analysis was based on limited empirical evidence to suggest that varied study measures of sleep disturbances resulted in substantial differences in results (Schønning et al., 2022; Scott et al., 2021). Third, the present study included a large age‐span (i.e., early childhood, middle childhood and adolescence) and we acknowledge that ACEs affect sleep differently at each developmental stage of the child (Schønning et al., 2022); we have conducted sensitivity analyses (i.e., exclusion of studies on early childhood) and determined that this limitation did not significantly bias our results. Fourth, the present study examined a saturated mediation model (i.e., included all possible pathways among variables) and we could not evaluate model fit indices. Fifth, we had used the beta estimation procedure for some studies (*k* = 8) and this approach may result in biased imputed correlation coefficients (Roth et al., 2018). Accordingly, we performed post‐hoc sensitivity analysis and observed stable model results; thus, we did not exclude these studies. Finally, we did not include unpublished studies and this could have led to some publication bias; however, current evidence suggests an unclear impact on meta‐analysis results and including unpublished data may not fully account for all hidden data (Schmucker et al., 2017).

### Future research directions and conclusion

The present study was focused on children and adolescents and it will be clinically useful to compare mediation models between children/adolescents and adult populations. This will enable clinicians to gain a comprehensive understanding on how ACEs and sleep disturbance impact psychopathology across the developmental trajectory. In addition to comparing between different populations, future research should also investigate the synergistic effects of sleep disturbance with other risk mechanisms as multiple risk mechanisms are involved in the pathway between ACEs and psychopathology (Miu et al., 2022). Future research should use MASEM to evaluate a parallel mediation model for sleep disturbance and emotion dysregulation in the relationship between ACEs and psychopathology. In conclusion, the present meta‐analytic results support the hypothesis that sleep disturbance is one underlying mechanism in the relationship between ACEs and psychopathology among children and adolescents. Further research is required to elucidate the complex pathways that lead to disorder in the aftermath of childhood adversities.

## AUTHOR CONTRIBUTIONS

**Jianlin Liu:** Conceptualization, Data curation, Formal analysis, Investigation, Methodology, Project administration, Resources, Software, Visualization, Writing – original draft, Writing – review & editing. **Wen Lin Teh:** Conceptualization, Data curation, Investigation, Methodology, Project administration, Resources, Writing – review & editing. **Rachel Hsiao Shen Tan:** Data curation, Investigation, Methodology, Project administration, Writing – review & editing. **Yoke Boon Tan:** Data curation, Investigation, Methodology, Project administration, Writing – review & editing. **Charmaine Tang:** Conceptualization, Investigation, Writing – review & editing. **Nisha Chandwani:** Conceptualization, Investigation, Writing – review & editing. **Mythily Subramaniam:** Conceptualization, Investigation, Methodology, Project administration, Resources, Supervision, Writing – review & editing.

## CONFLICT OF INTEREST STATEMENT

The authors declared that they have no competing or potential conflicts of interest.

## ETHICAL CONSIDERATIONS

This research was conducted in accordance with the principles embodied in the Declaration of Helsinki and in accordance with local statutory requirements. All the studies included in this review have been approved by their respective institutional or national research ethics committees and review boards.

## Supporting information

Supplementary Material

## Data Availability

The data and R code that support the findings of this study are available on Open Science Framework (OSF): https://osf.io/fbntw/?view_only=1f0510095d1e41a493d26db1afacdf8a.
