# Supplementary material for: Sleep disturbance as transdiagnostic mediator between adverse childhood experiences and psychopathology in children and adolescents: A structural equation modeling meta‐analysis
Source: JCPP Adv. 2023 Mar 23;3(3):e12156. doi: 10.1002/jcv2.12156 (PMC10501693; doi:10.1002/jcv2.12156)
Supplement: Supplementary file 1 — Supplementary Material [file JCV2-3-e12156-s001.docx]

**Supporting Information**

| Table S1. Search Strategy for MEDLINE (PubMed). | | |
| --- | --- | --- |
| Search number | Terms | Results |
| 1 | (((child[MeSH Terms]) OR (adolescent[MeSH Terms])) OR (minors[MeSH Terms])) OR (pediatrics[MeSH Terms]) | 3,292,349 |
| 2 | (((((child*) OR (teen*)) OR (adolescen*)) OR (minors)) OR (pediatric)) OR (paediatric) | 1,932,287 |
| 3 | (#1) OR (#2) | 3,850,781 |
| 4 | (sleep[MeSH Terms]) OR (sleep wake disorders[MeSH Terms]) | 162,113 |
| 5 | (((((((((sleep disorder*) OR (sleep disturbance*)) OR (sleep deprivation)) OR (sleep problem*)) OR (sleep difficult*)) | 76,751 |
| 6 | (#4) OR (#5) | 98,434 |
| 7 | ((violence[MeSH Terms]) OR (stress disorders, traumatic[MeSH Terms])) OR (adverse childhood experiences[MeSH Terms]) | 84,250 |
| 8 | ((((((maltreatment) OR (abuse)) OR (neglect)) OR (childhood trauma)) OR (childhood adversit*)) OR (adverse childhood experience* OR adverse childhood event*) | 86,769 |
| 9 | (#7) OR (#8) | 144,878 |
| 10 | ((#3) AND (#6)) AND (#9) | 859 |
| Filters: Abstract; Full text; Humans; English; MEDLINE, from 1/1/1994 - 23/5/2022 | | |

Table S2. Study characteristics of included studies.

| **S/N** | **Study** | **Country** | **Study Design** | **Type of sample^b^** | **Sample size (female)** | **Mean age (SD)** | **ACEs measure** | **Types of ACEs** | **Sleep disturbance measure** | **Types of sleep disturbance** | **Psychopathology measure** | **Types of psychopathology** |
| --- | --- | --- | --- | --- | --- | --- | --- | --- | --- | --- | --- | --- |
| 1 | Afifi et al., 2022 | Canada | Cross-sectional^a^ | Non-clinical | 1002 (518) | 15.3  Age range 14-17 years | CTQ  CEVQ  7 items adapted from the 2014 Ontario Child Health Survey, the Manitoba Youth Health Survey, and the National Crime Victimization Survey  Yes/No items | Emotional neglect; emotional abuse; exposure to verbal IPV; spanking; foster care or CPO contact; parental separation/divorce; household mental illness; household substance abuse; parental gambling; parental trouble with police; poverty; lives in unsafe community; peer victimization | General self-report questionnaire | Sleep duration | Yes/No and frequency items | Past 30-day substance use (cigarette, alcohol, cannabis) |
| 2 | Ahonen et al., 2007 | Barcelona | Cross-sectional | Non-clinical | 2727 (1349) | Age range 14-18 years | Items adapted from Health Behaviour in School-aged Children, Youth Risk Behavior Surveillance System, and Peer Harassment in School surveys | Bullying; perceived mistreatment/abuse | - | - | 6 items adapted from the Health Behaviour in School-aged Children (HBSC) survey  Yes/No items | Symptoms of negative mood states; use of substances |
| 3 | Alshekaili et al., 2020 | Oman | Cross-sectional | Not categorized | 34 (22) | < 12 years: 23 ≥ 12 years: 11 | Child and Adolescent Mental Health Services referrals | Child sexual abuse | Yes/No items | Disturbed sleep-wake cycles | CIDI  Yes/No items | Depressive symptoms |
| 4 | Anastasia et al., 2022 | Italy | Cross-sectional | Not categorized | 212 (91) | Age range 1-17 years | WAST | Witnessed violence (measured in the form of mothers' exposure to intimate partner violence) | General self-report questionnaire | Sleep disturbances | General self-report questionnaire | Anxiety/Aggressive symptoms |
| 5 | April-Sanders et al., 2021 | New York and Puerto Rico | Longitudinal | Non-clinical | 2491 (1224) | Sample 1: 7.11; Age range 5-9 years   Sample 2: 11.57; Age range 10-16 years | 11 items from validated scales | Parental loss; parental maltreatment; parental maladjustment; exposure to violence | DISC-4 | Trouble falling/staying asleep; daytime sleepiness; bad dreams/nightmares | - | - |
| 6 | Baddam et al., 2019 | Texas | Cross-sectional^a^ | Non-clinical | 752 (386) | 13.48 (1.02) Age range 12-14 years | CTQ  SLES | Emotional abuse; physical abuse; sexual abuse; emotional neglect; physical neglect; stressful life events in the past year | DOTS  YSR | Movement during sleep; regularity of sleep; sleep disturbances; sleep pressure | SCARED  MFQ-C | Anxiety symptoms; depressive symptoms |
| 7 | Bagley et al., 2016 | United States | Cross-sectional^a^ | Non-clinical | 252 (134) | 15.79 (0.81) | Neighborhood Walkability Scale  Community Experiences Questionnaire | Community violence | Actigraphy  School Sleep Habits Survey | Sleep minutes; sleep efficiency; long-wake episodes; sleep/wake problems; sleepiness | - | - |
| 8 | Bailey et al., 2005 | Michigan | Cross-sectional | Non-clinical | 268 (138) | 6.9 Age range 6.4-7.9 years | TISH  Parent report  Conflict Tactics Scale  Daily Hassles Test | Community violence; sexual abuse; physical abuse; domestic violence; family life stress | Levonn items | Difficulty sleeping | Levonn items  CBCL | Traumatic stress symptoms |
| 9 | Baniasad et al., 2016 | Iran | Cross-sectional | Non-clinical | 215 | Age range 13-15 years | CASRS | Emotional abuse; ignorance abuse; physical abuse; sexual abuse | GHQ | Insomnia symptoms | GHQ | Depressive symptoms |
| 10 | Bicanic et al., 2013 | Netherlands | Cross-sectional | Mixed | 89 | Sample 1: 16.1 (15.5-16.6)  Sample 2: 15.6 (15.1-16.2) | Psychotrauma Centre referrals | Single rape event | Salivary cortisol, DHEAS and DEX cortisol | Sleep duration | ADIS-C  CDI | Depression; externalizing problems |
| 11 | Boduszek et al., 2021 | Uganda and Jamaica | Cross-sectional | Non-clinical | 18700 (10401) | Sample 1: 14 (1.95); Age range 9-17 years  Sample 2: 13.74 (1.97); Age range 9-17 years | CVEQ | Corporal punishment; physical abuse; non-contact sexual abuse; contact sexual abuse; emotional abuse; physical neglect; medical neglect; emotional neglect | PROMIS | Sleep problems | Yes/No items  PROMIS | Suicidal ideation; suicidal attempt; non-suicidal self-injury; anxiety; depression |
| 12 | Bronstein et al., 2013 | London | Cross-sectional | Not categorized | 222 | 16.34 (1.03) | RATS | ACEs assessed as part of PTSD assessment | SHS | Sleeping patterns; nightmare frequency | RATS | PTSD |
| 13 | Brown et al., 2011 | New Orleans | Longitudinal | Non-clinical | 191 (86) | 11.5  Age range 8-15 years | Yes/No item | Exposure to hurricane and its aftermath | 2 items from RCADS | Sleep disturbances; fear of sleeping alone | PTSD-RI | PTSD symptoms |
| 14 | Caldwell et al., 2015 | United States | Cross-sectional | Not categorized | 21 (10) | Age range 3-4 years | PSI  PDS  BDI  BAI | Mother's psychological status (stress, PTSD, depression, anxiety) | Actigraphy  Sleep diary  CSHQ | Nocturnal sleep duration; time in bed; nocturnal sleep efficiency; sleep problems | - | - |
| 15 | Chae et al., 2021 | Korea | Cross-sectional^a^ | Non-clinical | 1276 (634) | Age range 13-15 years | Items | Child abuse experience (excessive discipline, physical abuse with no reason, physical abuse causing injury, verbal/emotional abuse) | Items | Sleep quality; sleep duration | Yes/No item | Depressive symptoms |
| 16 | Chang, 2019 | Taiwan | Longitudinal | Non-clinical | 2280 (1139) | Age 7-16 years | 14 items | Family dysfunction | PSQI | Sleep quality | - | - |
| 17 | Chemtob et al., 2008 | New York | Cross-sectional | Not categorized | 116 (57) | 3.9 (1.0) | Dichotomous index of exposure to high-intensity WTC events  TESI | Exposure to high-intensity WTC Attack-related events; exposure to other traumatic events | CBCL | Sleep problems | CBCL | Anxious/depressed behavioral symptoms |
| 18 | Chen et al., 2021 | China | Longitudinal | Not categorized | 707 (421) | 15.43 (0.65) | 4 items | Earthquake exposure (family members' death, missing, and/or injury; house damage; property loss; witness of tragic scenes) | PSQI | Sleep problems | PTSD-SS  PCL-5  SCARED  SCAARED  DSRSC  PHQ-9 | PTSD symptoms; anxiety symptoms; depressive symptoms |
| 19 | Chung et al., 2022 | Australia | Cross-sectional | Mixed | 63 (49) | Sample 1: 14.1 (1.4)  Sample 2: 13.7 (2.5) | Structured interviews  ELSQ | Bullying; physical abuse; sexual abuse; emotional abuse; neglect; parental separation; loss by separation; loss by death; family conflict; severe illness of a family member; domestic violence; birth complications; life-threatening/severe illness; war trauma; natural disasters | Structured interviews | Sleep issues (difficulties falling asleep, waking, unrefreshing sleep) | Structured interviews | Functional neurological disorder |
| 20 | Choquet et al., 1997 | France | Cross-sectional | Non-clinical | 183 (94) | 16.2 (2.02) | Questionnaire derived two other studies | Victims of violence; sexual assault | Multiple choice items | Sleep disorders (sleeping badly, nightmares, waking during the night) | Questionnaire derived two other studies  Multiple choice items | Depressive symptoms |
| 21 | Demirci et al., 2018 | Turkey | Cross-sectional | Not categorized | 85 (55) | Sample 1: 12.5 (3.4)  Sample 2: 13.9 (2.6)  Sample 3: 14.3 (2.3) | Assessed by Child and Adolescent Psychiatry Department | Child sexual abuse | PSQI  ISI | Sleep quality and quantity; frequency and severity of sleep disturbance | DEBQ  DERS | non-suicidal self-injury; emotional eating; difficulties in emotion regulation |
| 22 | Dirkzwager et al., 2006 | Netherlands | Longitudinal | Not categorized | 3576 (1676) | Sample 1: 9.17 (5.62)  Sample 2: 9.14 (5.46) | Electronic medical records of family practitioners | Fireworks disaster | ICPC | Sleep problems | - | - |
| 23 | Dubois-Comtois et al., 2016 | Canada | Cross-sectional | Not categorized | 25 | 60.24 months (18.70); Age range 3-7 years | Child Protection Services referrals | Neglect; physical abuse; sexual abuse; physical and sexual abuse | Your Child Sleep | Non-restorative sleep (difficulties waking-up in the morning, sleepiness after final morning awakening, tiredness during the day, and falling asleep during the day); poor sleep (difficulty falling asleep, difficulty getting back to sleep following an awakening, being anxious at night/afraid of darkness); parasomnia (body rocking, sleep bruxism, enuresis, bad dreams/nightmares, night terrors, and irregular breathing during sleep) | - | - |
| 24 | Edgardh et al., 2000 | Sweden | Cross-sectional | Non-clinical | 2153 (1243) | 17-year-old sample | Questionnaire | Sexual abuse | Questionnaire | Sleeping disturbances | Questionnaire | Eating disorders; suicidal thoughts and attempts |
| 25 | Eiset et al., 2020 | Denmark | Cross-sectional | Not categorized | 7210 (3100) | < 18 years | Clinical interview | Separation from family members; loss of family members; poverty and starvation; violence | Clinical interview | Sleeping problems | Clinical interview  The Child Scale (triage tool) | Anxiety symptoms; depressive symptoms |
| 26 | Fekkes et al., 2004 | Netherlands | Cross-sectional^a^ | Non-clinical | 2766 (1384) | 10.1 (1.1) | 1 item | Being bullied | Frequency item | Sleeping problems | KDVK | Anxiety symptoms; depression |
| 27 | Foss et al., 2021 | United States | Longitudinal | Non-clinical | 204 (204) | 17.89 (1.18) | CTQ | Childhood maltreatment (emotional abuse, emotional neglect, physical abuse, physical neglect, sexual abuse) | PSQI | Sleep quality | PDQ | Pregnancy-related worries, anxiety, and distress |
| 28 | Gartland et al., 2021 | Australia | Longitudinal | Non-clinical | 615 | Age range 1-10 years | CAS | Intimate partner violence (emotional abuse, physical abuse) | 1 item | Sleep problems | SDQ  SCAS  DAWBA | Emotional/behavioural difficulties; anxiety; probable psychiatric diagnosis |
| 29 | Greeson et al., 2014 | United States | Cross-sectional | Not categorized | 11028 (5776) | Sample 1: 4.2 (1.1)  Sample 2: 11.9 (3.4) | UCLA PTSD-RI | Trauma exposure (traumatic loss/separation/bereavement, domestic violence, impaired caregiver, emotional abuse, physical abuse, neglect, sexual abuse, community violence, sexual assault, school violence, other trauma, serious injury, physical assault, illness/medical trauma, interpersonal violence, natural disaster, kidnapping, forced displacement, war/terrorism/political violence outside United States, war/terrorism/political violence inside United States) | CBCL | Sleep problems | CBCL | Depressive symptoms; anxiety symptoms |
| 30 | Gregory et al., 2006 | New Zealand | Longitudinal | Non-clinical | 936 (458) | 18-year-old sample | MFES | Family conflict | Dichotomous scale  DIS | Childhood sleep problems; insomnia | DIS | Depression |
| 31 | Guo et al., 2018 | China | Cross-sectional^a^ | Non-clinical | 75715 (40775) | 16.6 (1.2) | CTQ | Childhood maltreatment (emotional abuse, emotional neglect, physical abuse, physical neglect, sexual abuse) | PSQI | Subjective sleep quality; sleep disturbances | Yes/No item | Suicide attempt |
| 32 | Hall Brown et al., 2016 | United States | Cross-sectional | Not categorized | 4043 (2238) | Sample 1: 10.0 (1.7)  Sample 2: 10.2 (1.7)  Sample 3: 15.2 (1.4)  Sample 4: 15.6 (1.4) | UCLA PTSD-RI | Lifetime trauma exposure | Clinical assessment and collateral information from multiple sources | Sleep disturbance | UCLA PTSD-RI | PTSD symptoms |
| 33 | Hall Brown et al., 2019 | United States | Cross-sectional | Not categorized | 147 (50) | 16.00 (1.24) | Yes/No items adapted from CDC ACEs questionnaire and TESI-C | Child maltreatment | FoSI-Short Form  ISI | Trauma-related nocturnal fears, concerns, and behaviours; insomnia | PC-PTSD | PTSD symptoms |
| 34 | Hambrick et al., 2018 | United States | Cross-sectional | Not categorized | 516 (246) | 10.3 (0.90) | 6 items  Items adapted from TISH | Physical abuse; sexual abuse; removal from a single parent household; exposure to community violence; number of caregiver transitions; number of school transitions | CBCL | Sleep problems | TSCC PTS | PTSD symptoms |
| 35 | Hash et al., 2019 | United States | Longitudinal | Not categorized | 247 (114) | Sample 1: 15.98 months (4.37)  Sample 2: 16.78 months (4.55) | Department of Health and Human Services records  Items from CLE, CES-D 2nd edition, DLC and investigator-developed | Potential maltreatment; removal from birth home; caregiver mental illness; caregiver incarceration; domestic violence; household substance use or abuse; caregiver divorce or separation; death of a family member or loved one; homelessness | 1 item from BISQ | Sleep problems | - | - |
| 36 | Hébert et al., 2017 | Montreal | Cross-sectional | Not categorized | 179 (148) | Sample: 4.67 (0.77) | Medical files  History of Victimization Form | Child sexual abuse; physical abuse; psychological abuse; neglect; exposure to interparental violence | CBCL-Preschool Version | Sleep problems | Child Dissociative Checklist | Dissociative symptoms |
| 37 | Heissel et al., 2018 | United States | Cross-sectional | Non-clinical | 82 (40) | 14.90 (1.87) | Geocoded file from city police department | violent community crime | Acti-watch-64 data  Self-report diaries  Salivary samples | Bedtime; sleep latency; wake time; sleep duration; bedtime cortisol; waking cortisol; cortisol awakening response | - | - |
| 38 | Hildenbrand et al., 2013 | United States | Cross-sectional | Non-clinical | 14782 (7018) | Age <15 to ≥ 18 years; 86% below 18 years | Dichotomized items | School violence; violence-related injury; bullying | Items | Sleep duration | - | - |
| 39 | Huang et al., 2021 | China | Cross-sectional | Not categorized | 490 (234) | 13.40 (1.38) | Revised Adverse Childhood Experiences Scale | Frequent peer victimization experiences; other ACEs | YSIS | Insomnia symptoms | PHQ-9  GAD-7 | Depressive symptoms; anxiety symptoms |
| 40 | Ji et al., 2019 | China | Cross-sectional^a^ | Non-clinical | 707 (342) | 13.16 (0.90) | CTSPC | Physical abuse | PSQI | Sleep quality | - | - |
| 41 | Johnson et al., 2022 | Texas | Cross-sectional | Non-clinical | 515 (263) | 17.13 (1.06) | Dichotomous items | Parental incarceration; living with someone who drinks too much alcohol; living with someone who uses illegal drugs; history of verbal abuse; history of physical abuse; history of sexual abuse; history of domestic violence in the home | 1 item | Inadequate sleep | Yes/No items | Depressive symptoms; anxiety symptoms; PTSD symptoms |
| 42 | Jones et al., 2021 | United States | Cross-sectional | Mixed | 20 | Sample 1: 14.67 (0.94)  Sample 2: 14.52 (0.95) | Local mental health facilities referrals  CTQ  SLES Adolescent Report | Sexual abuse; witnessing violence; traumatic death of loved one; accident; physical abuse | Actigraphy | Sleep disorders | - | - |
| 43 | Kamphhuis et al., 2008 | Gaza Strip and Galilee, Israel | Cross-sectional | Not categorized | 319 (145) | Sample 1: 10.85 (2.42)  Sample 2: 11.58 (3.04) | TEC | Night raids; beatings; witnessing beatings; detention; observed humiliation; imprisonment of family members | Dream diary  Rorschach inkblot method | Traumatic dream content | PSS | Psychological symptoms |
| 44 | King et al., 2021 | United States | Cross-sectional | Not categorized | 100 (77) | 15.4 (1.58) | Psychiatrist assessment  Patient's electronic medical record | Physical abuse; sexual abuse; witness to violence; severe neglect; bullying; developmental trauma | FOSI  PSQI | Fear of sleep; sleep disturbances; sleep quality | SITBI-SR | Suicide attempt |
| 45 | Kliewer et al., 2015 | United States | Longitudinal | Not categorized | 362 (185) | 12.45 (0.59) | SCECV | Community violence | SWBPS | Sleep problems | CRIES | Intrusive thoughts |
| 46 | Kliewer et al., 2019 | United States | Cross-sectional | Non-clinical | 107 (60) | 14.29 (1.17) | SCEV | Community violence | Health history  SWBPS | Obstructive sleep apnea; erratic sleep-wake behaviours | CDI | Depressive symptoms |
| 47 | Kshirsagar et al., 2007 | India | Cross-sectional | Non-clinical | 500 (312) | Age range 8-12 years | Semi-structured questionnaire | Bullying | Semi-structured questionnaire | Nightmares; disturbance in sleep | - | - |
| 48 | Laberge, L | Quebec | Cross-sectional^a^ | Non-clinical | 1353 (689) | Age range 6-16 years | FAI | Family adversity | Items | Parasomnias | SBQ | Anxiety symptoms |
| 49 | Lai et al., 2020 | Texas | Longitudinal | Not categorized | 269 (143) | Sample: 8.70 (0.95) | HURTE-R  LEC | Hurricane exposure; hurricane-related stressors; major life events | YSR | Sleep problems | UCLA PTSD-RI-Revised | PTSD symptoms |
| 50 | Lamers-Winkelman et al., 2012 | Netherlands | Cross-sectional | Not categorized | Sample 1 (child witnesses of IPV): 275 (129)  Sample 2 (children from general population): 903 (461) | Sample 1: 8.62 (1.70)  Sample 2: 9.12 (1.96) | Parent Report of Traumatic Event  Extensive caregiver interview  Referral to services for IPV  Reports/records from other agencies | Witnessing IPV; physical abuse; sexual abuse; emotional abuse; neglect | CBCL | Overtired; trouble sleeping; sleep less; sleep more; nightmares; wets bed | - | - |
| 51 | Langevin et al., 2019 | Canada | Cross-sectional | Not categorized | 315 (134) | 15.51 (1.04) | Assessment by nurses  Admission records | Physical abuse; sexual abuse | 4-item self-report scale | Poor sleep quality; difficulty falling asleep; frequent awakenings; frequent nightmares | Self-report scale | Mood symptoms; anxiety symptoms; ADHD symptoms |
| 52 | Langevin et al., 2017 | Canada | Longitudinal | Not categorized | Sample 1 (child protection clinic): 224 (191)  Sample 2 (general population): 83 (49) | 4.7 (0.8) | History of Victimization Questionnaire | Sexual abuse | CBCL (preschool) sleep subscale | Sleep problems | - | - |
| 53 | Lee et al., 2020 | South Korea | Longitudinal | Not categorized | 31 (19) | Graduated from high school (approximately 17-18 years) | ACEs-Q | Physical abuse or neglect, sexual abuse, emotional abuse or neglect, witnessing domestic violence, substance misuse or mental illness within the household, experiencing parental separation or divorce, or having an incarcerated household member, neighborhood violence | ISI | Insomnia | Inventory of Complicated Grief | Complicated grief |
| 54 | Lepore et al., 2013 | USA | Longitudinal | Not categorized | 498 (279) | 12.8 (0.44) | Survey of Children’s Exposure to Community Violence  Problem Behavior Frequency Scales (relational & physical victimization subscales) | Community violence; peer victimization | Sleep/Wake Behavior Problems | Erratic sleep-wake behaviors | Children's Depression Inventory Short Form  Children's Revised Impact of Events Scale (4-item intrusions subscale) | Depressive symptoms; intrusive symptoms |
| 55 | Lereya et al., 2017 | UK | Longitudinal | Non-clinical (preschool) | 6050 (3110) | 11.8  Age range 11-12 years | 2-item (mother-report) | Physical abuse; sexual abuse | 3-item (mother-report) | Nightmares; sleep maintenance problems; sleep onset problems | UK-CI-BPD | Bipolar disorder symptoms |
| 56 | Lyon et al., 2000 | USA | Cross-sectional | Not categorized | Sample 1 (suicide attempters): 38 (31)  Sample 2 (non-suicidal primary care patients): 76 (62) | Sample 1: 14.7  Sample 2: 14.9   Age range 12-17 years | Psychiatric Consultation Checklist | Death of parent; loss of significant other; suicide completion/attempt of family member or friend; actual or threatened separation from parental figure; abuse; neglect; parental substance abuse; parental history of depression; parental psychiatric history | Psychiatric Consultation Checklist | Insomnia | Psychiatric Consultation Checklist  Medical attention for suicide attempt | Suicide attempt |
| 57 | Malta et al., 2014 | Brazil | Cross-sectional | Non-clinical (school) | 109104 (56952) | Ninth grade students (approximately 14-15 years) | 1-item self-report | Bullying | 1-item self-report | Insomnia | - | - |
| 58 | Mansbach-Kleinfeld et al., 2015 | Israel | Cross-sectional | Non-clinical | 906 (443) | Age range 14-17 years | Self-report item | Sexual abuse | Self-report item | Difficulty falling asleep | DAWBA | Depression |
| 59 | Marie-Mitchell et al., 2020 | USA | Cross-sectional | Not categorized | 499 (245) | Age range 5-11 years | Whole Child Assessment | Emotional abuse; physical abuse; sexual abuse; neglect; parental separation; witnessing domestic violence; household member with drug/alcohol problem, mental illness, or incarceration | Whole Child Assessment | Sleep problems | Medical charts/diagnoses | Depression; anxiety; ADHD |
| 60 | Mayes et al., 2014 | USA | Cross-sectional | Clinical | 90 (87) | 13.8  Age range 7-18 years | PBS | Sexual abuse; physical abuse; peer victimization/bullying | PBS | Sleep disturbances (e.g., sleeps less than most other children, trouble falling asleep, wakes during the night, nightmares) | PBS | Suicidal ideation; suicide attempt |
| 61 | McGlinchey, E et al., 2015 | USA | Longitudinal | General population | Wave 2: 4834 (2519) | Wave 2: 16 | Self-report (yes/no) | Violence (fights that result in hospitalization) | Self-report | Late bedtime; late midsleep | Self-report | Emotional distress; alcohol abuse |
| 62 | McPhie et al., 2014 | Canada | Longitudinal | Not categorized | 73 (47) | 15.9 (1.06) | CTQ | Emotional abuse; physical abuse; emotional neglect; physical neglect; sexual abuse | 11-item self-report | Sleep disturbances | Brief Symptom Inventory (Global Severity Index) | Symptom dimensions: somatization, obsessive-compulsive, depression, anxiety, hostility, phobic anxiety, paranoid ideation, psychoticism |
| 63 | Mehtar et al., 2011 | Turkey | Cross-sectional | Clinical | 69 (16) | 11 years 7 months (3 years 3 months) | K-SADS-PL | Accidents/disasters; violence; physical abuse; sexual abuse | Trauma symptoms investigation form in Autistic Spectrum Disorders | Sleep disturbance | Structured interviews | ASD symptoms |
| 64 | Mello et al., 2016 | Brazil | Cross-sectional | Non-clinical (school) | 19660 | Ninth grade students (approximately 14-15 years) | 1-item self-report | Bullying | 1-item self-report | Insomnia | - | - |
| 65 | Merhy et al., 2021 | Lebanon | Cross-sectional | Non-clinical | 1801 (963) | 15.36 (1.15) | CASRS  Illinois bullying scale | Physical abuse; sexual abuse; psychological/ emotional abuse; neglect; bullying | Lebanese Insomnia Scale (LIS-8) | Insomnia | Lebanese Anxiety Scale | Anxiety symptoms |
| 66 | Mignot et al., 2018 | France | Cross-sectional | Non-clinical | 1719 (869) | 15-year-old sample | HBSC | Sexual abuse | HBSC | Quality of sleep (not satisfactory; difficulty falling asleep; nocturnal awakening; waking up too early; nightmares) | HBSC | Suicide attempts; suicidal ideation; self-mutilation |
| 67 | Muluk et al., 2015 | Turkey | Cross-sectional | Non-clinical (school) | 428 (244) | 14.16 (1.87) | Self-report items | Experiencing violence; exposure to explosion; intrafamilial violence | Self-report item | Sleep problems | - | - |
| 68 | Noel et al., 2018 | USA | Cross-sectional | Non-clinical | 97 (70) | 15.0 (2.1) | CPSS-5 | Witnessing death; physical illness or hospitalisation; sexual abuse; physical abuse; etc | Revised Adolescent Sleep-Wake Scale (rASWS) | Sleep quality | CPSS-5 | PTSD symptoms |
| 69 | Okada et al., 2018 | Japan | Cross-sectional | Not categorized | 273 (133) | 9.9 (3.2) | General self-report questionnaire | Abuse | Brief 19-item sleep questionnaire | Bedtime symptoms; sleep symptoms; waking symptoms; daytime sleepiness symptom | Questionnaire of emotional and behavioural problems | Autistic behaviour; attachment problems; ADHD behaviour; antisocial behaviour; depressive behaviour |
| 70 | Park et al., 2020 | Korea | Cross-sectional | Non-clinical | 737 (289) | 15.1 (1.4) | ETISR-SF  LTE-Q | Physical abuse; sexual abuse; emotional abuse; general trauma; serious illness or injury to self or close relative; death of relative; breaking off a steady relationship; serious problems with a close friend; becoming unemployed; being sacked from one’s job; a major financial crisis; problems with police and court appearance; something valuable being lost or stolen | ISI  Epworth Sleepiness Scale  School Sleep Habits Survey | Insomnia; daytime sleepiness; problems with sleep initiation and maintenance | Children's Depression Inventory  Revised Children's Manifest Anxiety Scale | Depressive symptoms; anxiety symptoms |
| 71 | Pavlova et al., 2020 | Canada | Longitudinal | Not categorized | 138 (104) | 14.29 | K-SADS-PL (PTSD scale) | DSM-5 Criterion A events | Actigraphy  Adolescent Sleep-Wake Scale  ISI | Sleep initiation; sleep maintenance; overall sleep quality; insomnia | K-SADS-PL (PTSD scale)  CPSS-5 | PTSD symptoms |
| 72 | Rojo-Wissar et al., 2021 | USA | Longitudinal | Not categorized | 817 (402) | 5.06 (0.02) | Parent-reported items | Parent substance abuse; parent disability; parent incarceration; family violence; parent separation/divorce; parent death; child abuse and neglect; poverty | Adolescent self-report | Total sleep time; social jetlag; trouble falling asleep; trouble staying asleep | Center for Epidemiological Studies Depression Scale  BSI-18 anxiety subscale | Depressive symptoms; anxiety symptoms |
| 73 | Sarigedik et al., 2021 | Turkey | Cross-sectional | Mixed | Sample 1 (females with PTSD related to sexual abuse): 40 (40)  Sample 2 (healthy female controls): 40 (40) | Sample 1: 15.29 (1.75)  Sample 2: 15.06 (1.66) | Exposure to sexual abuse | Sexual abuse | PSQI  Epworth Sleepiness Scale  ISI | Sleep disorders; daytime sleepiness; insomnia | CAPS-CA  KSADS-PL | PTSD symptoms |
| 74 | Schneiderman et al., 2018 | USA | Longitudinal | Not categorized | 385 (199) | 13.7 (1.42) | Child welfare case | Neglect; emotional abuse; physical abuse; sexual abuse | PSQI | Sleep duration; sleep disturbance | Children's Depressive Inventory  Youth Symptom Survey Checklist | Depressive symptoms; PTSD symptoms |
| 75 | Semenza et al., 2022 | USA | Cross-sectional | Non-clinical (school) | 9819 (5106) | Ninth grade students (approximately 14-15 years) | Self-report questions | Bullying victimization; family drug problems | 1-item self-report | Sleep duration | Self-report questions | Depressive symptoms |
| 76 | Setanu et al., 2021 | Romania | Cross-sectional | Non-clinical | 118 (79) | Age range 6-17 years | PSQ-9 (for fathers)  1 item on criticism of child | Parental depression; being criticised by parents | DSM-5 Parent/ Guardian-Rated Level 1 Cross-Cutting Symptom Measure, Child Age 6, 17 | Sleep disturbances | DSM-5 Parent/ Guardian-Rated Level 1 Cross-Cutting Symptom Measure, Child Age 6, 17 | Inattention symptoms; depressive symptoms; anxiety symptoms |
| 77 | Sharma et al., 2017 | Oceania | Cross-sectional | Non-clinical (school) | 4122 (2246) | ≤14 years: 2144 ≥15 years: 1893 | 1-item self-report | Bullying; injury | 1-item self-report | Insomnia | 1-item self-report | Suicidal ideation; suicide attempt |
| 78 | Shen et al., 2015 | China | Cross-sectional | Non-clinical (school) | 2283 (1256) | 14.22 (1.71) | Left-behind children status | Parents' migration / separation from parent(s); physical abuse; injury | Not reported | Difficulty falling asleep | Children's Depression Inventory (Chinese version)  Screen for Child Anxiety Related Emotional Disorders | Depressive symptoms; anxiety symptoms |
| 79 | Sterling et al., 2021 | USA | Cross-sectional | Non-clinical | 13370 (6490) | Age range 1-5 years | Modified ACES questionnaire | Physical abuse or neglect, sexual abuse, emotional abuse or neglect, witnessing domestic violence, substance misuse or mental illness within the household, experiencing parental separation or divorce, or having an incarcerated household member, foster care placement, food, housing or clothing insecurity, family separation, life-threatening illness, or neighborhood violence | Medical records | Sleep disorder | Medical records | Adjustment disorders; autism spectrum disorders |
| 80 | Swangpun et al., 2019 | Thailand | Cross-sectional | Non-clinical (school) | 3353 | 4.6 (1.03) | Scale for risk assessment for drug abuse among preschoolers | Abuse; neglect; family substance abuse; parents' changing marital status; conflict between parents; family poverty | Scale for risk assessment for drug abuse among preschoolers | Sleep problems | Self-designed scale | ADHD |
| 81 | Tang et al., 2018 | China | Cross-sectional | Not categorized | 5563 (2989) | 14.4 (1.98) | 8-item scale | Exposure to natural disaster (earthquake) | PSQI | Subjective sleep quality; sleep latency; sleep duration; habitual sleep efficiency; sleep disturbances, sleep medication use; daytime dysfunction | Children's Revised Impact of Event Scale  Short Mood and Feelings Questionnaire  Screen for Child Anxiety-Related Emotional Disorders | PTSD symptoms; depressive symptoms; anxiety symptoms |
| 82 | Turner et al., 2020 | Canada | Cross-sectional | Non-clinical | 2910 (1415) | Age range 14-17 years | Childhood Experiences of Violence Questionnaire  Survey questions designed for the National Longitudinal Study of Adolescent to Adult Health | Sexual abuse; physical abuse; exposure to intimate partner violence; emotional maltreatment; physical neglect | Self-report items | Time it takes to fall asleep; waking during the night; hours of sleep on weekdays and weekends | - | - |
| 83 | Umlauf et al., 2015 | USA | Cross-sectional | Non-clinical | 263 (129) | Age range 14-15 years | Two self-report items | Exposure to violence | Two self-report items  Sleep-50 inventory | Sleep disorder symptoms (insomnia, apnea, nightmares, restless legs syndrome or periodic limb movement disorder); daytime sleepiness | - | - |
| 84 | Usami et al., 2013 | Japan | Cross-sectional | Not categorized | 11692 (5733) | 10.9 (2.7) | Self-report questionnaire | Earthquake/tsunami exposure | Sleep questionnaire | Waking and sleep onset times | Post Traumatic Stress Symptoms for Children 15-items (PTSSC-15) | PTSD symptoms |
| 85 | Usta et al., 2010 | Lebanon | Cross-sectional | Non-clinical | 1028 (472) | 11.89 (1.67) | International Child Abuse Screening Tool | Sexual abuse | Trauma Symptom Checklist - sleep disturbance subscale | Sleep disturbances | Trauma Symptom Checklist for Children | Depression symptoms; anxiety symptoms; PTSD symptoms |
| 86 | Vanaelst et al., 2012 | Europe | Cross-sectional^a^ | Non-clinical | 4066 (2045) | 7.91 (1.82) | IDEFICS (Identification and prevention of Dietary- and lifestyle-induced health EFfects In Children and infantS) questionnaire | Negative life events; chronic familial or social adversities | IDEFICS (Identification and prevention of Dietary- and lifestyle-induced health EFfects In Children and infantS) questionnaire | Difficulties falling asleep | Strengths and Difficulties Questionnaire | Emotional/behavioral difficulties |
| 87 | Wamser-Nanney et al., 2018 | USA | Cross-sectional | Not categorized | 276 (175) | 10.88 (3.39) | Intake assessment | Complex trauma; sexual abuse; physical abuse; domestic violence; emotional abuse; neglect; death of a loved one; community violence; serious illness; serious injury/accident; natural disaster; school violence; kidnapping; war/terrorism | Child Behavior Checklist 6-18 | Nightmares; overtired; sleeps more than other kids; sleeps less than other kids; walks/ talks in sleep; and trouble sleeping | - | - |
| 88 | Wang et al., 2022 | China | Cross-sectional | Non-clinical | 17023 (8178) | 6.17 (0.29) | Parent-reported items | Physical abuse; emotional abuse; sexual abuse; physical neglect | Children's Sleep Habits Questionnaire | Bedtime resistance; sleep onset delay; sleep duration; sleep anxiety; night wakings; parasomnias; sleep disordered breathing; daytime sleepiness | Strengths and Difficulties Questionnaire | Emotional/behavioral difficulties |
| 89 | Wells et al., 1995 | USA | Cross-sectional | Not categorized | 204 | 7 Age range 2-11 years | Exposure to sexual abuse | Sexual abuse | Structured Interview for Signs Associated with Sexual Abuse (SASA) | Difficulty sleeping; sleeping more than usual; nightmares | - | - |
| 90 | Wittmann et al., 2010 | Switzerland | Longitudinal | Not categorized | 32 (12) | 11.9 (2.4) | Medical reports | Road traffic accident | Clinician-Administered PTSD Scale, Child and Adolescent Version - nightmare frequency scale | Nightmares | Children's Depression Inventory  Clinician-Administered PTSD Scale, Child and Adolescent Version | PTSD symptoms; depressive symptoms |
| 91 | Yen et al., 2010 | Taiwan | Cross-sectional | Non-clinical (school) | 6406 (3369) | 14.8 (1.8) | School Bullying Experience Questionnaire (Chinese version) | Bullying | Athens Insomnia Scale (Taiwanese version) | Insomnia | Center for Epidemiological Studies Depression Scale (Mandarin Chinese version)  Multidimensional Anxiety Scale for Children (Taiwanese version)  Social Phobia Inventory  Attention Deficit/Hyperactivity Disorder Self-Rated Scale  Kiddie-Schedule for Affective Disorders and Schizophrenia - suicidality scale  CRAFFT alcohol abuse screening test | Depression symptoms; general anxiety symptoms; social phobia symptoms; ADHD symptoms; suicidality; alcohol abuse |
| 92 | Yuksel et al., 2022 | USA | Cross-sectional | Not categorized | Sample 1 (insomnia): 47 (31)  Sample 2 (controls): 48 (28) | Sample 1: 17.7 (0.9)  Sample 2: 18.2 (1.0) | ACEs-Q | Physical abuse or neglect, sexual abuse, emotional abuse or neglect, witnessing domestic violence, substance misuse or mental illness within the household, experiencing parental separation or divorce, or having an incarcerated household member, neighborhood violence | ISI  PSQI  Morning-eveningness questionnaire  Cleveland Adolescent Sleepiness Questionnaire | Insomnia; sleep quality and disturbances; circadian typology; daytime sleepiness | BDI-2  State-Trait Anxiety Inventory (STAI-Y2) | Depressive symptoms; anxiety symptoms |
| 93 | Zhang et al., 2015 | China | Longitudinal | Not categorized | 2299 (1199) | 11.75 (2.56) | - | - | PHQ-15 | Trouble sleeping | Children's Revised Impact of Event Scale | PTSD symptoms |
| 94 | Zhang et al., 2022 | USA | Longitudinal | Non-clinical | 124 (64) | 12.89 (0.79) | CTQ | Threat (emotional abuse, physical abuse); deprivation (emotional neglect; physical neglect) | PSQI | Sleep quality | Covid-19 Adolescent Symptom & Psychological Experience Questionnaire | Negative affect |
| 95 | Zhou et al., 2022 | China | Cross-sectional | Non-clinical | 6122 (3134) | 16.12 (1.44) | 4-item self-report | Physical abuse by parents; sibling aggression; peer bullying | PSQI  Nightmare Distress Questionnaire (Chinese version)  Chinese Adolescent Daytime Sleepiness Scale  Multidimensional Fatigue Inventory | Sleep quality; nightmare distress; daytime sleepiness; fatigue | Mini-International Neuropsychiatric Interview - suicide risk module | Suicide risk |
| 96 | Zhou et al., 2017 | China | Longitudinal | Not categorized | 746 (414) | 15.02 (1.63) | Trauma exposure questionnaire | Witnessing or hearing about the death, injury, or entrapment of parents, friends, teachers, or others | Child Behavior Problems Questionnaire | Sleep problems | Child PTSD Symptom Scale | PTSD symptoms |
| 97 | Insana et al., 2014 | USA | Cross-sectional | Not categorized | Sample 1 (children of IPV exposed mothers): 40 (23)  Sample 2 (children of mothers from community): 60 (10) | Sample 1: 9.53 (1.93)  Sample 2: 9.2 (1.6) | O'Leary-Porter Scale  Mothers experienced IPV | Exposure to IPV | Child Behavior Checklist 6-18 | Nightmares; sleep walking/talking; bed wetting; sleeping less; sleeping more; trouble sleeping | - | - |
| 98 | Secrist et al., 2019 | USA | Cross-sectional | Non-clinical | 60 (22) | Age 6-11 | Trauma History Screen | Stressful or traumatic life events | Children's Sleep Habits Questionnaire- Abbreviated  Trauma Related Nightmare Survey - Modified Version  Nightmare Distress Questionnaire - Modified | Sleep behaviours; nightmare distress | Child Dissociative Checklist  Revised Children's Manifest Anxiety Scale | Dissociative behaviours; anxiety symptoms |

| Table S3. Pooled correlations (below the diagonal) and proportion of between-study heterogeneity *I^2^* (above the diagonal) based on Stage 1 random-effect analyses. | | | |
| --- | --- | --- | --- |
|  | ACEs | Sleep Disturbance | Psychopathology |
| ACEs | 1 | 0.97 | 0.98 |
| Sleep Disturbance | 0.21*** | 1 | 0.98 |
| Psychopathology | 0.24*** | 0.29*** | 1 |
| Note: ACEs = Adverse Childhood Experiences; ****p* < .001 | | | |

| Table S4. Pooled correlations (below the diagonal) and proportion of between-study heterogeneity *I^2^* (above the diagonal) based on subgroup (study design) Stage 1 random-effect analyses. | | | | | | | |
| --- | --- | --- | --- | --- | --- | --- | --- |
|  | Cross-sectional subgroup (*k* = 74) | | |  | Longitudinal subgroup (*k* = 24) | | |
|  | ACEs | Sleep Disturbance | Psychopathology |  | ACEs | Sleep Disturbance | Psychopathology |
| ACEs | 1 | 0.98 | 0.98 | ACEs | 1 | 0.89 | 0.91 |
| Sleep Disturbance | 0.22 | 1 | 0.99 | Sleep Disturbance | 0.17 | 1 | 0.94 |
| Psychopathology | 0.27 | 0.28 | 1 | Psychopathology | 0.15 | 0.29 | 1 |
| Note: ACEs = Adverse Childhood Experiences; ****p* < .001 | | | | | | | |

| Table S5. Pooled correlations (below the diagonal) and proportion of between-study heterogeneity *I^2^* (above the diagonal) based on subgroup (geographical difference) Stage 1 random-effect analyses. | | | | | | | |
| --- | --- | --- | --- | --- | --- | --- | --- |
|  | Non-Western subgroup (*k* = 30) | | |  | Western subgroup (*k* = 68) | | |
|  | ACEs | Sleep Disturbance | Psychopathology |  | ACEs | Sleep Disturbance | Psychopathology |
| ACEs | 1 | 0.96 | 0.99 | ACEs | 1 | 0.97 | 0.97 |
| Sleep Disturbance | 0.17 | 1 | 0.99 | Sleep Disturbance | 0.22 | 1 | 0.97 |
| Psychopathology | 0.24 | 0.29 | 1 | Psychopathology | 0.23 | 0.27 | 1 |
| Note: ACEs = Adverse Childhood Experiences; ****p* < .001 | | | | | | | |

| Table S6. Parameter estimates and 95% confidence intervals based on subgroup (study design) Stage 2 random-effect analyses. | | | | | | | |
| --- | --- | --- | --- | --- | --- | --- | --- |
|  | Cross-sectional subgroup (*k* = 74) | | | Longitudinal subgroup (*k* = 24) | | | *χ^2^* (df = 3) |
|  | Estimate | LLCI | ULCI | Estimate | LLCI | ULCI | 9.71 |
| *^β^*ACEs 🡪 Sleep disturbance | 0.22 | 0.18 | 0.27 | 0.17 | 0.12 | 0.22 |  |
| *^β^*Sleep disturbance 🡪 Psychopathology | 0.23 | 0.17 | 0.30 | 0.27 | 0.20 | 0.34 |  |
| *^β^*ACEs 🡪 Psychopathology | 0.21 | 0.15 | 0.27 | 0.10 | 0.04 | 0.17 |  |
| Indirect effect | 0.05 | 0.03 | 0.07 | 0.04 | 0.03 | 0.07 |  |
| Note: ACEs = Adverse Childhood Experiences; LLCI = lower level confidence interval; ULCI = upper level confidence interval | | | | | | | |

| Table S7. Parameter estimates and 95% confidence intervals based on subgroup (geographical difference) Stage 2 random-effect analyses. | | | | | | | |
| --- | --- | --- | --- | --- | --- | --- | --- |
|  | Non-Western subgroup (*k* = 30) | | | Western subgroup (*k* = 68) | | | *χ^2^* (df = 3) |
|  | Estimate | LLCI | ULCI | Estimate | LLCI | ULCI | 1.95 |
| *^β^*ACEs 🡪 Sleep disturbance | 0.17 | 0.12 | 0.22 | 0.17 | 0.12 | 0.22 |  |
| *^β^*Sleep disturbance 🡪 Psychopathology | 0.26 | 0.17 | 0.34 | 0.26 | 0.17 | 0.34 |  |
| *^β^*ACEs 🡪 Psychopathology | 0.20 | 0.10 | 0.30 | 0.20 | 0.10 | 0.30 |  |
| Indirect effect | 0.04 | 0.02 | 0.06 | 0.05 | 0.02 | 0.06 |  |
| Note: ACEs = Adverse Childhood Experiences; LLCI = lower level confidence interval; ULCI = upper level confidence interval | | | | | | | |

Table S8. Quality assessments.

| **S/N** | **Study** | **Q1** | **Q2** | **Q3** | **Q4** | **Q5** | **Q6** | **Q7** | **Q8** | **Q9** | **Q10** | **Q11** | **Q12** | **Q13** | **Q14** |
| --- | --- | --- | --- | --- | --- | --- | --- | --- | --- | --- | --- | --- | --- | --- | --- |
| 1 | Afifi et al., 2022 | Yes | Yes | Yes | Yes | No | No | No | Yes | Yes | No | Yes | No | NA | Yes |
| 2 | Ahonen et al., 2007 | Yes | Yes | Yes | Yes | No | No | No | Yes | Yes | No | Yes | No | NA | Yes |
| 3 | Alshekaili et al., 2020 | Yes | Yes | Yes | Yes | No | No | No | Yes | Yes | No | Yes | No | NA | No |
| 4 | Anastasia et al., 2022 | Yes | Yes | Yes | Yes | No | No | No | Yes | Yes | No | No | No | NA | No |
| 5 | April-Sanders et al., 2021 | Yes | Yes | Yes | Yes | No | Yes | Yes | Yes | Yes | No | Yes | No | Yes | Yes |
| 6 | Baddam et al., 2019 | Yes | Yes | Yes | Yes | No | No | No | Yes | Yes | No | Yes | No | NA | Yes |
| 7 | Bagley et al., 2016 | Yes | Yes | Yes | Yes | No | No | No | Yes | Yes | No | Yes | No | NA | Yes |
| 8 | Bailey et al., 2005 | Yes | Yes | Yes | Yes | No | No | No | Yes | Yes | No | Yes | No | NA | Yes |
| 9 | Baniasad et al., 2016 | No | Yes | Yes | No | Yes | No | No | Yes | Yes | No | Yes | No | NA | No |
| 10 | Biacnic et al., 2013 | Yes | Yes | Yes | Yes | No | No | No | No | No | No | Yes | No | NA | Yes |
| 11 | Boduszek et al., 2021 | Yes | No | Yes | No | No | No | No | Yes | Yes | No | Yes | No | NA | Yes |
| 12 | Bronstein et al., 2013 | Yes | Yes | Yes | Yes | No | No | No | No | No | No | Yes | No | NA | No |
| 13 | Brown et al., 2011 | Yes | Yes | Yes | No | No | Yes | Yes | No | Yes | No | Yes | No | Yes | Yes |
| 14 | Caldwell et al., 2015 | Yes | Yes | Yes | Yes | No | No | No | Yes | Yes | No | Yes | No | NA | No |
| 15 | Chae et al., 2021 | Yes | Yes | Yes | No | No | No | No | Yes | Yes | No | Yes | No | NA | Yes |
| 16 | Chang et al., 2019 | Yes | Yes | Yes | No | No | Yes | Yes | Yes | Yes | Yes | Yes | No | Yes | Yes |
| 17 | Chemtob et al., 2008 | Yes | Yes | Yes | Yes | No | No | No | Yes | Yes | No | Yes | No | NA | Yes |
| 18 | Chen et al., 2021 | Yes | Yes | Yes | No | No | Yes | Yes | Yes | Yes | No | Yes | No | No | Yes |
| 19 | Chung et al., 2022 | Yes | Yes | Yes | Yes | No | No | No | Yes | Yes | No | Yes | No | NA | No |
| 20 | Choquet et al., 1997 | Yes | Yes | Yes | No | No | No | No | No | No | No | Yes | No | NA | Yes |
| 21 | Demirci et al., 2018 | Yes | Yes | Yes | Yes | No | No | No | No | No | No | No | No | NA | Yes |
| 22 | Dirkzwager et al., 2006 | Yes | Yes | Yes | No | No | Yes | Yes | No | Yes | No | Yes | No | Yes | No |
| 23 | Dubois-Comtois et al., 2016 | Yes | Yes | Yes | Yes | No | No | No | Yes | Yes | No | Yes | No | NA | Yes |
| 24 | Edgardh et al., 2000 | No | Yes | No | No | Yes | No | No | Yes | Yes | No | No | No | NA | No |
| 25 | Eiset et al., 2020 | Yes | Yes | Yes | Yes | No | No | No | No | No | No | No | No | NA | Yes |
| 26 | Fekkes et al., 2004 | Yes | Yes | Yes | No | No | No | No | Yes | Yes | No | Yes | No | NA | Yes |
| 27 | Foss et al., 2021 | Yes | Yes | Yes | Yes | No | Yes | Yes | Yes | Yes | No | Yes | No | Yes | Yes |
| 28 | Gartland et al., 2021 | Yes | Yes | Yes | Yes | No | Yes | Yes | Yes | Yes | Yes | Yes | No | Yes | Yes |
| 29 | Greeson et al., 2014 | Yes | Yes | Yes | Yes | No | No | No | Yes | Yes | No | Yes | No | NA | Yes |
| 30 | Gregory et al., 2006 | Yes | Yes | Yes | No | No | Yes | Yes | Yes | Yes | Yes | Yes | No | Yes | Yes |
| 31 | Guo et al., 2018 | Yes | Yes | Yes | No | No | No | No | Yes | Yes | No | Yes | No | NA | Yes |
| 32 | Hall Brown et al., 2016 | Yes | Yes | Yes | Yes | No | No | No | Yes | Yes | No | Yes | No | NA | Yes |
| 33 | Hall Brown et al., 2019 | Yes | Yes | No | Yes | No | No | No | Yes | Yes | No | Yes | No | NA | Yes |
| 34 | Hambrick et al., 2018 | Yes | Yes | Yes | Yes | No | No | No | Yes | Yes | No | Yes | No | NA | Yes |
| 35 | Hash et al., 2019 | Yes | Yes | Yes | Yes | No | Yes | Yes | Yes | Yes | Yes | Yes | Yes | Yes | Yes |
| 36 | Hébert et al., 2017 | Yes | No | Yes | Yes | No | No | No | Yes | Yes | No | Yes | No | NA | Yes |
| 37 | Heissel et al., 2018 | Yes | Yes | Yes | No | No | No | No | Yes | Yes | No | Yes | No | NA | Yes |
| 38 | Hildenbrand et al., 2013 | Yes | Yes | Yes | No | No | No | No | Yes | Yes | No | Yes | No | NA | Yes |
| 39 | Huang et al., 2021 | Yes | Yes | Yes | No | No | No | No | Yes | Yes | No | Yes | No | NA | Yes |
| 40 | Ji et al., 2019 | Yes | Yes | Yes | No | No | No | No | Yes | Yes | No | Yes | No | NA | Yes |
| 41 | Johnson et al., 2022 | Yes | Yes | Yes | No | No | No | No | Yes | Yes | No | Yes | No | NA | No |
| 42 | Jones et al., 2021 | Yes | No | Yes | Yes | No | No | No | Yes | Yes | No | Yes | No | NA | Yes |
| 43 | Kamphhuis et al., 2008 | Yes | Yes | Yes | No | No | No | No | Yes | Yes | No | Yes | No | NA | Yes |
| 44 | King et al., 2021 | Yes | Yes | Yes | No | No | No | No | Yes | Yes | No | Yes | No | NA | Yes |
| 45 | Kliewer et al., 2015 | Yes | Yes | Yes | No | No | Yes | Yes | Yes | Yes | No | Yes | No | Yes | Yes |
| 46 | Kliewer et al., 2019 | Yes | Yes | Yes | Yes | No | No | No | Yes | Yes | No | Yes | No | NA | Yes |
| 47 | Kshirsagar et al., 2007 | Yes | No | Yes | No | Yes | No | No | Yes | No | No | No | No | NA | No |
| 48 | Laberge et al., 2000 | Yes | No | Yes | No | No | No | No | Yes | Yes | No | Yes | No | NA | No |
| 49 | Lai et al., 2020 | Yes | Yes | Yes | Yes | No | Yes | Yes | Yes | Yes | No | Yes | No | Yes | Yes |
| 50 | Lamers-Winkelman et al., 2012 | Yes | Yes | Yes | Yes | No | No | No | Yes | Yes | No | Yes | No | NA | Yes |
| 51 | Langevin et al., 2019 | Yes | Yes | Yes | No | No | No | No | Yes | No | No | No | No | NA | Yes |
| 52 | Langevin et al., 2017 | Yes | No | No | Yes | No | Yes | No | No | Yes | No | Yes | No | No | Yes |
| 53 | Lee et al., 2020 | Yes | Yes | Yes | No | No | Yes | Yes | No | Yes | No | Yes | No | No | Yes |
| 54 | Lepore et al., 2013 | Yes | Yes | Yes | No | No | Yes | No | Yes | Yes | No | Yes | No | No | Yes |
| 55 | Lereya et al., 2017 | Yes | Yes | No | Yes | No | Yes | Yes | No | No | Yes | No | No | Yes | Yes |
| 56 | Lyon et al., 2000 | Yes | Yes | No | No | No | No | No | Yes | Yes | No | Yes | No | NA | Yes |
| 57 | Malta et al., 2014 | Yes | Yes | Yes | No | No | No | No | No | No | No | No | No | NA | Yes |
| 58 | Mansbach-Kleinfeld et al., 2015 | Yes | Yes | Yes | No | Yes | No | No | No | No | No | No | No | NA | Yes |
| 59 | Marie-Mitchell et al., 2020 | Yes | Yes | Yes | No | No | No | No | Yes | Yes | No | Yes | No | NA | Yes |
| 60 | Mayes et al., 2014 | Yes | Yes | Yes | No | No | No | No | Yes | Yes | No | Yes | No | NA | Yes |
| 61 | McGlinchey et al., 2015 | Yes | Yes | No | No | No | Yes | Yes | Yes | Yes | Yes | No | No | Yes | Yes |
| 62 | McPhie et al., 2014 | Yes | Yes | Yes | Yes | No | No | No | Yes | Yes | No | Yes | No | Yes | Yes |
| 63 | Mehtar et al., 2011 | Yes | Yes | Yes | Yes | No | No | No | No | Yes | No | Yes | No | NA | No |
| 64 | Mello et al., 2016 | Yes | Yes | Yes | No | No | No | No | No | No | No | Yes | No | NA | Yes |
| 65 | Merhy et al., 2021 | Yes | Yes | Yes | Yes | Yes | No | No | Yes | Yes | No | Yes | No | NA | Yes |
| 66 | Mignot et al., 2018 | Yes | Yes | No | No | No | No | No | No | Yes | No | Yes | No | NA | Yes |
| 67 | Muluk et al., 2015 | Yes | Yes | Yes | Yes | No | No | No | Yes | No | No | No | No | NA | No |
| 68 | Noel et al., 2018 | Yes | Yes | Yes | Yes | Yes | No | No | No | Yes | No | Yes | No | NA | Yes |
| 69 | Okada et al., 2018 | Yes | Yes | Yes | Yes | No | No | No | Yes | Yes | No | Yes | No | NA | Yes |
| 70 | Park et al., 2020 | Yes | Yes | Yes | No | No | No | No | No | Yes | No | Yes | No | NA | Yes |
| 71 | Pavlova et al., 2020 | Yes | Yes | Yes | Yes | No | No | No | Yes | Yes | Yes | Yes | No | Yes | Yes |
| 72 | Rojo-Wissar et al., 2021 | Yes | Yes | No | No | No | Yes | Yes | Yes | Yes | Yes | Yes | No | Yes | Yes |
| 73 | Sarigedik et al., 2021 | Yes | Yes | Yes | Yes | No | No | No | No | Yes | No | Yes | No | NA | Yes |
| 74 | Schneiderman et al., 2018 | Yes | Yes | Yes | Yes | No | Yes | Yes | Yes | Yes | Yes | Yes | No | Yes | Yes |
| 75 | Semenza et al., 2022 | Yes | Yes | Yes | No | No | No | No | Yes | No | No | No | No | NA | Yes |
| 76 | Setanu et al., 2021 | Yes | Yes | No | Yes | No | No | No | Yes | No | No | Yes | No | NA | Yes |
| 77 | Sharma et al., 2017 | Yes | Yes | Yes | No | No | No | No | Yes | No | No | No | No | NA | Yes |
| 78 | Shen et al., 2015 | Yes | Yes | Yes | Yes | No | No | No | No | No | No | No | No | NA | Yes |
| 79 | Sterling et al., 2021 | Yes | Yes | Yes | No | No | No | No | Yes | Yes | No | No | No | NA | Yes |
| 80 | Swangpun et al., 2019 | Yes | Yes | Yes | No | No | No | No | Yes | No | No | No | No | NA | No |
| 81 | Tang et al., 2018 | Yes | Yes | No | No | No | No | No | Yes | Yes | No | Yes | No | NA | Yes |
| 82 | Turner et al., 2020 | Yes | Yes | No | Yes | No | No | No | Yes | Yes | No | No | No | NA | Yes |
| 83 | Umlauf et al., 2015 | Yes | Yes | No | Yes | No | No | No | Yes | No | No | Yes | No | NA | Yes |
| 84 | Usami et al., 2013 | Yes | Yes | Yes | No | No | No | No | Yes | Yes | No | Yes | No | NA | No |
| 85 | Usta et al., 2010 | Yes | Yes | No | No | Yes | No | No | Yes | Yes | No | Yes | No | NA | Yes |
| 86 | Vanaelst et al., 2012 | Yes | Yes | Yes | No | No | No | No | Yes | Yes | No | Yes | No | NA | Yes |
| 87 | Wamser-Nanney et al., 2018 | Yes | Yes | No | Yes | No | No | No | Yes | Yes | No | Yes | No | NA | Yes |
| 88 | Wang et al., 2022 | Yes | Yes | Yes | No | No | No | No | Yes | No | No | Yes | No | NA | Yes |
| 89 | Wells et al., 1995 | Yes | Yes | No | No | No | No | No | No | Yes | No | Yes | No | NA | Yes |
| 90 | Wittmann et al., 2010 | Yes | Yes | No | Yes | No | Yes | Yes | Yes | Yes | Yes | Yes | No | Yes | Yes |
| 91 | Yen et al., 2010 | Yes | Yes | Yes | No | No | No | No | Yes | Yes | No | Yes | No | NA | Yes |
| 92 | Yuksel et al., 2022 | Yes | Yes | Yes | Yes | No | No | No | Yes | Yes | No | Yes | No | NA | Yes |
| 93 | Zhang et al., 2015 | Yes | Yes | No | No | No | Yes | No | Yes | Yes | Yes | Yes | No | No | Yes |
| 94 | Zhang et al., 2022 | Yes | No | No | Yes | No | Yes | No | Yes | Yes | Yes | Yes | No | Yes | Yes |
| 95 | Zhou et al., 2022 | Yes | No | No | Yes | No | No | No | Yes | No | No | Yes | No | NA | Yes |
| 96 | Zhou et al., 2017 | Yes | Yes | Yes | Yes | No | Yes | No | Yes | Yes | Yes | Yes | No | No | Yes |
| 97 | Insana et al., 2014 | Yes | Yes | No | Yes | No | No | No | Yes | Yes | No | Yes | No | NA | Yes |
| 98 | Secrist et al., 2019 | Yes | Yes | No | Yes | Yes | No | No | Yes | Yes | No | Yes | No | NA | Yes |

Note: Q1 = Clear research question or objective?; Q2 = Study population clearly defined?; Q3 = Response rate at least 50%?; Q4 = Clear inclusion and exclusion criteria?; Q5 = Power calculation reported?; Q6 = Was ACEs/sleep measured prior to the outcome (sleep disturbance or psychopathology)?: Q7 = Was the timeframe sufficient so that one could reasonably expect to see an association between exposure and outcome if it existed?; Q8 = Did the study examine different levels of ACEs/sleep as related to the outcome (e.g., categories of ACEs, or ACEs measured as continuous variable)?; Q9 = Were the ACEs/sleep measure clearly defined, valid, reliable, and implemented consistently across all study participants?; Q10 = Was exposure (ACEs/sleep) assessed more than once over time?; Q11 = Were the outcome measures (dependent variables) clearly defined, valid, reliable, and implemented consistently across all study participants?; Q12 = Were the outcome assessors blinded to the exposure status of participants?; Q13 = Was loss to follow-up after baseline 20% or less?; Q14 = Were key potential confounding variables measured and adjusted statistically for their impact on the relationship between ACEs and outcome(s)?; NA = not applicable.

**References of included articles (*k* = 98)**

Afifi, T. O., Taillieu, T., Salmon, S., Stewart-Tufescu, A., Struck, S., Fortier, J., MacMillan, H. L., Sareen, J., Tonmyr, L., & Katz, L. Y. (2022). Protective Factors for Decreasing Nicotine, Alcohol, and Cannabis Use Among Adolescents with a History of Adverse Childhood Experiences (ACEs). *International Journal of Mental Health and Addiction*. <https://doi.org/10.1007/s11469-021-00720-x>

Ahonen, E. Q., Nebot, M., & Giménez, E. (2007). Negative mood states and related factors in a sample of adolescent secondary-school students in Barcelona (Spain). *Gaceta Sanitaria*, *21*(1), 43–52. <https://doi.org/10.1157/13099120>

Alshekaili, M., Alkalbani, Y., Hassan, W., Alsulimani, F., Alkasbi, S., Chan, M. F., & Al-Adawi, S. (2020). Characteristic and psychosocial consequences of sexually abused children referred to a tertiary care facility in Oman: Sentinel study. *Heliyon*, *6*(1), e03150. <https://doi.org/10.1016/j.heliyon.2019.e03150>

Anastasia, F., Wiel, L. C., Giangreco, M., Morabito, G., Romito, P., Amaddeo, A., Barbi, E., & Germani, C. (2022). Prevalence of children witnessed violence in a pediatric emergency department. *European Journal of Pediatrics*, *181*(7), 2695–2703. <https://doi.org/10.1007/s00431-022-04474-z>

April-Sanders, A., Duarte, C. S., Wang, S., McGlinchey, E., Alcántara, C., Bird, H., Canino, G., & Suglia, S. F. (2020). Childhood Adversity and Sleep Disturbances: Longitudinal Results in Puerto Rican Children. *International Journal of Behavioral Medicine*, *28*(1), 107–115. <https://doi.org/10.1007/s12529-020-09873-w>

Baddam, Olvera, Canapari, Crowley, & Williamson. (2019). Childhood Trauma and Stressful Life Events Are Independently Associated with Sleep Disturbances in Adolescents. *Behavioral Sciences*, *9*(10), 108. <https://doi.org/10.3390/bs9100108>

Bagley, E. J., Tu, K. M., Buckhalt, J. A., & El-Sheikh, M. (2016). Community violence concerns and adolescent sleep. *Sleep Health*, *2*(1), 57–62. <https://doi.org/10.1016/j.sleh.2015.12.006>

Bailey, B. N., Delaney-Black, V., Hannigan, J. H., Ager, J., Sokol, R. J., & Covington, C. Y. (2005). Somatic Complaints in Children and Community Violence Exposure. *Journal of Developmental & Behavioral Pediatrics*, *26*(5), 341–348. <https://doi.org/10.1097/00004703-200510000-00001>

Baniasad, M.H., Noghani, F., Gerami, M., & Sadeghi, N. (2016). ‌Comparison of mental health in students with and without experience of child abuse. Acta Medica Mediterranea, 32, 2115.

Bicanic, I. A. E., Postma, R. M., Sinnema, G., De Roos, C., Olff, M., Van Wesel, F., & Van de Putte, E. M. (2013). Salivary cortisol and dehydroepiandrosterone sulfate in adolescent rape victims with post traumatic stress disorder. *Psychoneuroendocrinology*, *38*(3), 408–415. <https://doi.org/10.1016/j.psyneuen.2012.06.015>

Boduszek, D., Debowska, A., Ochen, E. A., Fray, C., Nanfuka, E. K., Powell-Booth, K., Turyomurugyendo, F., Nelson, K., Harvey, R., Willmott, D., & Mason, S. J. (2021). Prevalence and correlates of non-suicidal self-injury, suicidal ideation, and suicide attempt among children and adolescents: Findings from Uganda and Jamaica. Journal of Affective Disorders, 283, 172–178. <https://doi.org/10.1016/j.jad.2021.01.063>

Bronstein, I., & Montgomery, P. (2013). Sleeping Patterns of Afghan Unaccompanied Asylum-Seeking Adolescents: A Large Observational Study. *PLoS ONE*, *8*(2), e56156. <https://doi.org/10.1371/journal.pone.0056156>

Brown, T. H., Mellman, T. A., Alfano, C. A., & Weems, C. F. (2011). Sleep fears, sleep disturbance, and PTSD symptoms in minority youth exposed to Hurricane Katrina. *Journal of Traumatic Stress*, *24*(5), 575–580. <https://doi.org/10.1002/jts.20680>

Caldwell, B. A., & Redeker, N. S. (2014). Maternal Stress and Psychological Status and Sleep in Minority Preschool Children. *Public Health Nursing*, *32*(2), 101–111. <https://doi.org/10.1111/phn.12104>

Chae, W., Jang, J., Park, E.-C., & Jang, S.-I. (2021). Changes in child abuse experience associated to sleep quality: results of the Korean Children & Youth Panel Survey. *BMC Public Health*, *21*(1). <https://doi.org/10.1186/s12889-021-11309-3>

Chang, L.-Y., Wu, C.-C., Yen, L.-L., & Chang, H.-Y. (2019). The effects of family dysfunction trajectories during childhood and early adolescence on sleep quality during late adolescence: Resilience as a mediator. *Social Science & Medicine*, *222*, 162–170. <https://doi.org/10.1016/j.socscimed.2019.01.010>

Chemtob, C. M., Nomura, Y., & Abramovitz, R. A. (2008). Impact of Conjoined Exposure to the World Trade Center Attacks and to Other Traumatic Events on the Behavioral Problems of Preschool Children. *Archives of Pediatrics & Adolescent Medicine*, *162*(2), 126. <https://doi.org/10.1001/archpediatrics.2007.36>

Chen, X.-Y., Shi, X., Zhou, Y., Chen, H., Ma, Y., Wang, T., & Fan, F. (2021). Change patterns of sleep problems predict mental health problems among adolescents: A 10-year cohort study of Chinese Wenchuan earthquake. Journal of Affective Disorders, 287, 138-144. <https://doi.org/10.1016/j.jad.2021.02.080>

Chung, J., Mukerji, S., & Kozlowska, K. (2022). Cortisol and α-amylase awakening response in children and adolescents with functional neurological (conversion) disorder. *Australian & New Zealand Journal of Psychiatry*, 000486742210825. <https://doi.org/10.1177/00048674221082520>

Choquet, M., Darves-Bornoz, J.-M., Ledoux, S., Manfredi, R., & Hassler, C. (1997). Self-reported health and behavioral problems among adolescent victims of rape in France: Results of a cross-sectional survey. *Child Abuse & Neglect*, *21*(9), 823–832. <https://doi.org/10.1016/s0145-2134(97)00044-6>

Demirci, E. (2018). Non suicidal self-injury, emotional eating and insomnia after child sexual abuse: Are those symptoms related to emotion regulation? Journal of Forensic and Legal Medicine, 53, 17–21. <https://doi.org/10.1016/j.jflm.2017.10.012>

Dirkzwager, A. J. E., Kerssens, J. J., & Yzermans, C. J. (2006). Health Problems in Children and Adolescents Before and After a Man-made Disaster. *Journal of the American Academy of Child & Adolescent Psychiatry*, *45*(1), 94–103. <https://doi.org/10.1097/01.chi.0000186402.05465.f7>

Dubois-Comtois, K., Cyr, C., Pennestri, M.-H., & Godbout, R. (2016). Poor Quality of Sleep in Foster Children Relates to Maltreatment and Placement Conditions. SAGE Open, 6(4), 215824401666955. <https://doi.org/10.1177/2158244016669551>

Edgardh, K., & Ormstad, K. (2000). Prevalence and characteristics of sexual abuse in a national sample of Swedish seventeen-year-old boys and girls. *Acta Paediatrica*, *89*(3), 310–319. <https://pubmed.ncbi.nlm.nih.gov/10772279/>

Eiset, A. H., Loua, A. S., Kruse, A., & Norredam, M. (2020). The health status of newly arrived asylum-seeking minors in Denmark: a nationwide register-based study. *International Journal of Public Health*, *65*(9), 1763–1772. <https://doi.org/10.1007/s00038-020-01501-4>

Fekkes, M., Pijpers, F. I. M., & Verloove-Vanhorick, S. Pauline. (2004). Bullying behavior and associations with psychosomatic complaints and depression in victims. *The Journal of Pediatrics*, *144*(1), 17–22. <https://doi.org/10.1016/j.jpeds.2003.09.025>

Foss, S., Gustafsson, H. C., Berry, O. O., Hipwell, A. E., Werner, E. A., Peterson, B. S., & Monk, C. (2021). Associations between childhood maltreatment, poor sleep, and prenatal distress in pregnant adolescents. *Development and Psychopathology*, 1–10. <https://doi.org/10.1017/s0954579420002163>

Gartland, D., Conway, L. J., Giallo, R., Mensah, F. K., Cook, F., Hegarty, K., Herrman, H., Nicholson, J., Reilly, S., Hiscock, H., Sciberras, E., & Brown, S. J. (2021). Intimate partner violence and child outcomes at age 10: a pregnancy cohort. *Archives of Disease in Childhood*, archdischild-2020-320321. <https://doi.org/10.1136/archdischild-2020-320321>

Greeson, J. K. P., Briggs, E. C., Layne, C. M., Belcher, H. M. E., Ostrowski, S. A., Kim, S., Lee, R. C., Vivrette, R. L., Pynoos, R. S., & Fairbank, J. A. (2013). Traumatic Childhood Experiences in the 21st Century. *Journal of Interpersonal Violence*, *29*(3), 536–556. <https://doi.org/10.1177/0886260513505217>

Gregory, A. M., Caspi, A., Moffitt, T. E., & Poulton, R. (2006). Family Conflict in Childhood: A Predictor of Later Insomnia. *Sleep*, *29*(8), 1063–1067. <https://doi.org/10.1093/sleep/29.8.1063>

Guo, L., Wang, W., Gao, X., Huang, G., Li, P., & Lu, C. (2018). Associations of Childhood Maltreatment with Single and Multiple Suicide Attempts among Older Chinese Adolescents. The Journal of Pediatrics, 196, 244-250.e1. <https://doi.org/10.1016/j.jpeds.2018.01.032>

Hall Brown, T. S., Belcher, H. M. E., Accardo, J., Minhas, R., & Briggs, E. C. (2016). Trauma exposure and sleep disturbance in a sample of youth from the National Child Traumatic Stress Network Core Data Set. *Sleep Health*, *2*(2), 123–128. <https://doi.org/10.1016/j.sleh.2016.03.001>

Hall Brown, T. S., Garcia, E., Akeeb, A., Lynch-Jiles, A. C., White, D., & Young, M. (2019). Adolescent Nocturnal Fears: a psychometric evaluation of the fear of sleep inventory (FoSI). *Behavioral Sleep Medicine*, *17*(6), 721–728. <https://doi.org/10.1080/15402002.2018.1469495>

Hambrick, E. P., Rubens, S. L., Brawner, T. W., & Taussig, H. N. (2017). Do sleep problems mediate the link between adverse childhood experiences and delinquency in preadolescent children in foster care? Journal of Child Psychology and Psychiatry, 59(2), 140–149. <https://doi.org/10.1111/jcpp.12802>

Hash, J. B., Oxford, M. L., Fleming, C. B., Ward, T. M., Spieker, S. J., & Lohr, M. J. (2019). Impact of a home visiting program on sleep problems among young children experiencing adversity. *Child Abuse & Neglect*, *89*, 143–154. <https://doi.org/10.1016/j.chiabu.2018.12.016>

Hébert, M., Langevin, R., Guidi, E., Bernard-Bonnin, A. C., & Allard-Dansereau, C. (2016). Sleep problems and dissociation in preschool victims of sexual abuse. *Journal of Trauma & Dissociation*, 1–15. <https://doi.org/10.1080/15299732.2016.1240739>

Heissel, J. A., Sharkey, P. T., Torrats-Espinosa, G., Grant, K., & Adam, E. K. (2017). Violence and Vigilance: The Acute Effects of Community Violent Crime on Sleep and Cortisol. *Child Development*, *89*(4), e323–e331. <https://doi.org/10.1111/cdev.12889>

Hildenbrand, A. K., Daly, B. P., Nicholls, E., Brooks-Holliday, S., & Kloss, J. D. (2013). Increased Risk for School Violence-Related Behaviors Among Adolescents With Insufficient Sleep. *Journal of School Health*, *83*(6), 408–414. <https://doi.org/10.1111/josh.12044>

Huang, L., Liang, K., Jiang, W., Huang, Q., Gong, N., & Chi, X. (2021). Prevalence and Correlates of Mental Health Problems among Chinese Adolescents with Frequent Peer Victimization Experiences. *Children*, *8*(5), 403. <https://doi.org/10.3390/children8050403>

Insana, S.P., Foley, K.P., Montgomery-Downs, H.E., Kolko, D.J., & McNeil, C.B. (2014). Children exposed to intimate partner violence demonstrate disturbed sleep and impaired functional outcomes. *Psychological Trauma: Theory, Research, Practice, and Policy,* 6(3), 290-298. https://doi.org/10.1037/a0033108

Ji, X., Cui, N., & Liu, J. (2019). Using propensity score matching with doses in observational studies: An example from a child physical abuse and sleep quality study. *Research in Nursing & Health*, *42*(6), 436–445. <https://doi.org/10.1002/nur.21991>

Johnson, K. E., Hoskote, A. R., Rolin, D. G., & Kesler, S. R. (2021). Correlates of mental health problems among students in Texas alternative high schools and school‐level efforts to address mental health. *Research in Nursing & Health*. <https://doi.org/10.1002/nur.22197>

Jones, S., Castelnovo, A., Riedner, B., Flaherty, B., Prehn‐Kristensen, A., Benca, R., Tononi, G., & Herringa, R. (2021). Sleep and emotion processing in paediatric posttraumatic stress disorder: A pilot investigation. *Journal of Sleep Research*, *30*(4). <https://doi.org/10.1111/jsr.13261>

Kamphuis, J. H., Tuin, N., Timmermans, M., & Punamäki, R.-L. (2008). Extending the Rorschach Trauma Content Index and Aggression Indexes to Dream Narratives of Children Exposed to Enduring Violence: An Exploratory Study. *Journal of Personality Assessment*, *90*(6), 578–584. <https://doi.org/10.1080/00223890802388558>

King, C. D., Joyce, V. W., Nash, C. C., Buonopane, R. J., Black, J. M., Zuromski, K. L., & Millner, A. J. (2021). Fear of sleep and sleep quality mediate the relationship between trauma exposure and suicide attempt in adolescents. *Journal of Psychiatric Research*, *135*, 243–247. <https://doi.org/10.1016/j.jpsychires.2021.01.026>

Kliewer, W., & Lepore, S. J. (2014). Exposure to Violence, Social Cognitive Processing, and Sleep Problems in Urban Adolescents. *Journal of Youth and Adolescence*, *44*(2), 507–517. <https://doi.org/10.1007/s10964-014-0184-x>

Kliewer, W., Robins, J. L., & Borre, A. (2019). Community Violence Exposure, Sleep Disruption, and Insulin Resistance in Low-Income Urban Adolescents. *International Journal of Behavioral Medicine*, *26*(4), 437–442. <https://doi.org/10.1007/s12529-019-09801-7>

Kshirsagar, V. Y., Agarwal, R., & Bavdekar, S. B. (2007). Bullying in schools: prevalence and short-term impact. *Indian Pediatrics*, *44*(1), 25–28. <https://pubmed.ncbi.nlm.nih.gov/17277427/>

Laberge, L., Tremblay, R. E., Vitaro, F., Montplaisir, J., & PhD, Crcp. (2000). Development of Parasomnias From Childhood to Early Adolescence. *Pediatrics*, *106*(1), 67–74. <https://doi.org/10.1542/peds.106.1.67>

Lai, B. S., La Greca, A. M., Colgan, C. A., Herge, W., Chan, S., Medzhitova, J., Short, M., & Auslander, B. (2020). Sleep Problems and Posttraumatic Stress: Children Exposed to a Natural Disaster. *Journal of Pediatric Psychology*, *45*(9), 1016–1026. <https://doi.org/10.1093/jpepsy/jsaa061>

Lamers-Winkelman, F., Schipper, J. C. D., & Oosterman, M. (2012). Children’s physical health complaints after exposure to intimate partner violence. *British Journal of Health Psychology*, *17*(4), 771–784. <https://doi.org/10.1111/j.2044-8287.2012.02072.x>

Langevin, R., Hébert, M., Bergeron, S. J., Duchesne, M., Lambert, Y., Chartrand, R., & Frappier, J.-Y. (2019). Sleep problems and interpersonal violence in youth in care under the Quebec Child Welfare Society. *Sleep Medicine*, *56*, 52–56. <https://doi.org/10.1016/j.sleep.2018.11.003>

Langevin, R., Hébert, M., Guidi, E., Bernard-Bonnin, A.-C., & Allard-Dansereau, C. (2017). Sleep problems over a year in sexually abused preschoolers. *Paediatrics & Child Health*, *22*(5), 273–276. <https://doi.org/10.1093/pch/pxx077>

Lee, S. H., Seo, Y. E., Kim, K.-B., Noh, J.-W., & Kim, E. J. (2020). Long-term risks of complicated grief and insomnia in student survivors of the Sewol ferry disaster in South Korea: A four-year observational follow-up study. *Ethiopian Journal of Health Development*, *34*(3). <https://www.ajol.info/index.php/ejhd/article/view/198520>

Lepore, S. J., & Kliewer, W. (2013). Violence Exposure, Sleep Disturbance, and Poor Academic Performance in Middle School. *Journal of Abnormal Child Psychology*, *41*(8), 1179–1189. <https://doi.org/10.1007/s10802-013-9709-0>

Lereya, S. T., Winsper, C., Tang, N. K. Y., & Wolke, D. (2016). Sleep Problems in Childhood and Borderline Personality Disorder Symptoms in Early Adolescence. *Journal of Abnormal Child Psychology*, *45*(1), 193–206. <https://doi.org/10.1007/s10802-016-0158-4>

Lyon, M. E., Benoit, M., O’Donnell, R. M., Getson, P. R., Silber, T., & Walsh, T. (2000). Assessing African American adolescents’ risk for suicide attempts: attachment theory. *Adolescence*, *35*(137), 121–134. <https://pubmed.ncbi.nlm.nih.gov/10841301/>

Malta, D. C., Prado, R. R. do, Dias, A. J. R., Mello, F. C. M., Silva, M. A. I., Costa, M. R. da, & Caiaffa, W. T. (2014). Bullying and associated factors among Brazilian adolescents: analysis of the National Adolescent School-based Health Survey (PeNSE 2012). *Revista Brasileira de Epidemiologia*, *17*(suppl 1), 131–145. <https://doi.org/10.1590/1809-4503201400050011>

Mansbach-Kleinfeld, I., Ifrah, A., Apter, A., & Farbstein, I. (2015). Child sexual abuse as reported by Israeli adolescents: Social and health related correlates. *Child Abuse & Neglect*, *40*, 68–80. <https://doi.org/10.1016/j.chiabu.2014.11.014>

Marie-Mitchell, A., Watkins, H. B. R., Copado, I. A., & Distelberg, B. (2020). Use of the Whole Child Assessment to identify children at risk of poor outcomes. *Child Abuse & Neglect*, *104*, 104489. <https://doi.org/10.1016/j.chiabu.2020.104489>

Mayes, S. D., Fernandez-Mendoza, J., Baweja, R., Calhoun, S., Mahr, F., Aggarwal, R., & Arnold, M. (2014). Correlates of Suicide Ideation and Attempts in Children and Adolescents With Eating Disorders. *Eating Disorders*, *22*(4), 352–366. <https://doi.org/10.1080/10640266.2014.915694>

McGlinchey, E. L., & Harvey, A. G. (2014). Risk Behaviors and Negative Health Outcomes for Adolescents with Late Bedtimes. *Journal of Youth and Adolescence*, *44*(2), 478–488. <https://doi.org/10.1007/s10964-014-0110-2>

McPhie, M. L., Weiss, J. A., & Wekerle, C. (2014). Psychological distress as a mediator of the relationship between childhood maltreatment and sleep quality in adolescence: Results from the Maltreatment and Adolescent Pathways (MAP) Longitudinal Study. *Child Abuse & Neglect*, *38*(12), 2044–2052. <https://doi.org/10.1016/j.chiabu.2014.07.009>

Mehtar, M., & Mukaddes, N. M. (2011). Posttraumatic Stress Disorder in individuals with diagnosis of Autistic Spectrum Disorders. *Research in Autism Spectrum Disorders*, *5*(1), 539–546. <https://doi.org/10.1016/j.rasd.2010.06.020>

Mello, F. C. M., Malta, D. C., Prado, R. R. do, Farias, M. S., Alencastro, L. C. da S., & Silva, M. A. I. (2016). Bullying e fatores associados em adolescentes da Região Sudeste segundo a Pesquisa Nacional de Saúde do Escolar. *Revista Brasileira de Epidemiologia*, *19*(4), 866–877. <https://doi.org/10.1590/1980-5497201600040015>

Merhy, G., Azzi, V., Salameh, P., Obeid, S., & Hallit, S. (2021). Anxiety among Lebanese adolescents: scale validation and correlates. *BMC Pediatrics*, *21*(1). <https://doi.org/10.1186/s12887-021-02763-4>

Mignot, S., Fritel, X., Loreal, M., Binder, P., Roux, M.-T., Gicquel, L., & Ingrand, P. (2018). Identifying teenage sexual abuse victims by questions on their daily lives. *Child Abuse & Neglect*, *85*, 127–136. <https://doi.org/10.1016/j.chiabu.2018.07.027>

Muluk, N. B., Bulbul, S. F., Turğut, M., & Ağirtaş, G. (2015). Sleep Problems of Adolescents: A Detailed Survey. *Ear, Nose & Throat Journal*, *94*(6), E4–E11. <https://doi.org/10.1177/014556131509400614>

Noel, M., Vinall, J., Tomfohr-Madsen, L., Holley, A. L., Wilson, A. C., & Palermo, T. M. (2018). Sleep Mediates the Association Between PTSD Symptoms and Chronic Pain in Youth. *The Journal of Pain*, *19*(1), 67–75. <https://doi.org/10.1016/j.jpain.2017.09.002>

Okada, M., Otaga, M., Tsutsui, T., Tachimori, H., Kitamura, S., Higuchi, S., & Mishima, K. (2018). Association of sleep with emotional and behavioral problems among abused children and adolescents admitted to residential care facilities in Japan. *PLOS ONE*, *13*(6), e0198123. <https://doi.org/10.1371/journal.pone.0198123>

Park, E-Jin., Kim, S.-Y., Kim, Y., Sung, D., Kim, B., Hyun, Y., Jung, K.-I., Lee, S.-Y., Kim, H., Park, S., Kim, B.-N., & Park, M.-H. (2021). The Relationship between Adverse Childhood Experiences and Sleep Problems among Adolescent Students: Mediation by Depression or Anxiety. *International Journal of Environmental Research and Public Health*, *18*(1). <https://doi.org/10.3390/ijerph18010236>

Pavlova, M., Kopala-Sibley, D. C., Nania, C., Mychasiuk, R., Christensen, J., McPeak, A., Tomfohr-Madsen, L., Katz, J., Palermo, T. M., & Noel, M. (2020). Sleep disturbance underlies the co-occurrence of trauma and pediatric chronic pain: a longitudinal examination. *Pain*, *161*(4), 821–830. <https://doi.org/10.1097/j.pain.0000000000001769>

Rojo-Wissar, D. M., Sosnowski, D. W., Ingram, M. M., Jackson, C. L., Maher, B. S., Alfano, C. A., Meltzer, L. J., & Spira, A. P. (2021). Associations of adverse childhood experiences with adolescent total sleep time, social jetlag, and insomnia symptoms. *Sleep Medicine*, *88*, 104–115. <https://doi.org/10.1016/j.sleep.2021.10.019>

Sarigedik, E., & Yurteri, N. (2021). Evaluation of sleep quality and quality of life in female adolescents with post-traumatic stress disorder related to sexual abuse. *Psychiatry and Clinical Psychopharmacology, 31*(1), 90-97.

Schneiderman, J. U., Ji, J., Susman, E. J., & Negriff, S. (2018). Longitudinal Relationship Between Mental Health Symptoms and Sleep Disturbances and Duration in Maltreated and Comparison Adolescents. *Journal of Adolescent Health*, *63*(1), 74–80. <https://doi.org/10.1016/j.jadohealth.2018.01.011>

Secrist, M.E., Dalenberg, C.J., & Gevirtz, R. (2018). Contributing factors predicting nightmares in children: Trauma, anxiety, dissociation, and emotion regulation. *Psychological Trauma: Theory, Research, Practice, and Policy, 11*(1), 114-121.

Semenza, D. C., Meldrum, R. C., Testa, A., & Jackson, D. B. (2021). Sleep duration, depressive symptoms, and digital self‐harm among adolescents. *Child and Adolescent Mental Health*. <https://doi.org/10.1111/camh.12457>

Seteanu, S.L., & Giosan, C. (2021). Adverse childhood experiences in fathers and the consequences in their children. Professional Psychology: Research and Practice, 52(1), 80-89. <https://doi.org/10.1037/pro0000360>

Sharma, B., Lee, T. H., & Nam, E. W. (2017). Loneliness, Insomnia and Suicidal Behavior among School-Going Adolescents in Western Pacific Island Countries: Role of Violence and Injury. *International Journal of Environmental Research and Public Health*, *14*(7), 791. <https://doi.org/10.3390/ijerph14070791>

Shen, M., Gao, J., Liang, Z., Wang, Y., Du, Y., & Stallones, L. (2015). Parental migration patterns and risk of depression and anxiety disorder among rural children aged 10–18 years in China: a cross-sectional study. *BMJ Open*, *5*(12), e007802. <https://doi.org/10.1136/bmjopen-2015-007802>

Sterling, S., Chi, F., Lin, J., Padalkar, P., Vinayagasundaram, U., Iturralde, E., Young-Wolff, K., Metz, V. E., Herz, A., Negusse, R., Jackson-Morris, M., & Espinas, P. (2021). Physical, Mental Health and Developmental Conditions, and Sociodemographic Characteristics Associated With Adverse Childhood Experiences Among Young Children in Pediatric Primary Care. *Journal of Pediatric Health Care*, *35*(5), 491–499. <https://doi.org/10.1016/j.pedhc.2021.04.009>

Swangpun, K., Kanato, M., & Leyatikul, P. (2019). The Risk of Drug Abuse among Preschool Students in Phuket, Thailand. *Iranian Journal of Public Health*, *48*(3), 451–457. <https://www.ncbi.nlm.nih.gov/pmc/articles/PMC6570815/>

Tang, W., Lu, Y., Yang, Y., & Xu, J. (2018). An epidemiologic study of self-reported sleep problems in a large sample of adolescent earthquake survivors: The effects of age, gender, exposure, and psychopathology. *Journal of Psychosomatic Research*, *113*, 22–29. <https://doi.org/10.1016/j.jpsychores.2018.07.006>

Turner, S., Menzies, C., Fortier, J., Garces, I., Struck, S., Taillieu, T., Georgiades, K., & Afifi, T. O. (2020). Child maltreatment and sleep problems among adolescents in Ontario: A cross sectional study. *Child Abuse & Neglect*, *99*, 104309. <https://doi.org/10.1016/j.chiabu.2019.104309>

Umlauf, M. G., Bolland, A. C., Bolland, K. A., Tomek, S., & Bolland, J. M. (2014). The Effects of Age, Gender, Hopelessness, and Exposure to Violence on Sleep Disorder Symptoms and Daytime Sleepiness Among Adolescents in Impoverished Neighborhoods. *Journal of Youth and Adolescence*, *44*(2), 518–542. <https://doi.org/10.1007/s10964-014-0160-5>

Usami, M., Iwadare, Y., Kodaira, M., Watanabe, K., Aoki, M., Katsumi, C., Matsuda, K., Makino, K., Iijima, S., Harada, M., Tanaka, H., Sasaki, Y., Tanaka, T., Ushijima, H., & Saito, K. (2013). Sleep Duration among Children 8 Months after the 2011 Japan Earthquake and Tsunami. *PLoS ONE*, *8*(5), e65398. <https://doi.org/10.1371/journal.pone.0065398>

Usta, J., & Farver, J. (2010). Child sexual abuse in Lebanon during war and peace. *Child: Care, Health and Development*, *36*(3), 361–368. <https://doi.org/10.1111/j.1365-2214.2010.01082.x>

Vanaelst, B., De Vriendt, T., Ahrens, W., Bammann, K., Hadjigeorgiou, C., Konstabel, K., Lissner, L., Michels, N., Molnar, D., Moreno, L. A., Reisch, L., Siani, A., Sioen, I., & De Henauw, S. (2012). Prevalence of psychosomatic and emotional symptoms in European school-aged children and its relationship with childhood adversities: results from the IDEFICS study. *European Child & Adolescent Psychiatry*, *21*(5), 253–265. <https://doi.org/10.1007/s00787-012-0258-9>

Wamser-Nanney, R., & Chesher, R. E. (2018). Trauma characteristics and sleep impairment among trauma-exposed children. *Child Abuse & Neglect*, *76*, 469–479. <https://doi.org/10.1016/j.chiabu.2017.11.020>

Wang, Z., Li, W., Cui, N., Sun, X., Rong, T., Deng, Y., Meng, M., Shan, W., Zhang, Y., Ordway, M., Jiang, F., & Wang, G. (2022). The association between child maltreatment and sleep disturbances among preschoolers. *Child Abuse & Neglect*, *127*, 105525. <https://doi.org/10.1016/j.chiabu.2022.105525>

Wells, R. D., McCann, J., Adams, J., Voris, J., & Ensign, J. (1995). Emotional, behavioral, and physical symptoms reported by parents of sexually abused, nonabused, and allegedly abused prepubescent females. *Child Abuse & Neglect*, *19*(2), 155–163. <https://doi.org/10.1016/0145-2134(94)00113-9>

Wittmann, L., Zehnder, D., Schredl, M., Jenni, O. G., & Landolt, M. A. (2010). Posttraumatic nightmares and psychopathology in children after road traffic accidents. *Journal of Traumatic Stress*, *23*(2), 232-239. <https://doi.org/10.1002/jts.20514>

Yen, C.-F., Yang, P., Wang, P.-W., Lin, H.-C., Liu, T.-L., Wu, Y.-Y., & Tang, T.-C. (2014). Association between school bullying levels/types and mental health problems among Taiwanese adolescents. *Comprehensive Psychiatry*, *55*(3), 405–413. <https://doi.org/10.1016/j.comppsych.2013.06.001>

Yuksel, D., Kiss, O., Prouty, D. E., Baker, F. C., & de Zambotti, M. (2022). Clinical characterization of insomnia in adolescents – an integrated approach to psychopathology. *Sleep Medicine*, *93*, 26–38. <https://doi.org/10.1016/j.sleep.2022.03.010>

Zhang, J., Zhu, S., Du, C., & Zhang, Y. (2015). Posttraumatic Stress Disorder and Somatic Symptoms Among Child and Adolescent Survivors Following the Lushan Earthquake in China: A Six-Month Longitudinal Study. *Journal of Psychosomatic Research*, 79, 100-106. <https://pubmed.ncbi.nlm.nih.gov/26080620/>

Zhang, L., Cui, Z., Sasser, J., Carvalho, C., & Oshri, A. (2022). Family stress during the pandemic worsens the effect of adverse parenting on adolescent sleep quality. *Child Abuse & Neglect*, *123*, 105390. <https://doi.org/10.1016/j.chiabu.2021.105390>

Zhou, S.-J., Wang, L.-L., Wang, T.-T., Wang, J.-Q., & Chen, J.-X. (2022). Associations between Experienced Aggression, Poor Sleep, and Suicide Risk among Chinese Adolescents. *Sleep*. <https://doi.org/10.1093/sleep/zsac048>

Zhou, X., Wu, X., Chen, Q., & Zhen, R. (2017). Why did adolescents have sleep problems after earthquakes? Understanding the role of traumatic exposure, fear, and PTSD. *Scandinavian Journal of Psychology*, *58*(3), 221–227. <https://doi.org/10.1111/sjop.12366>

**
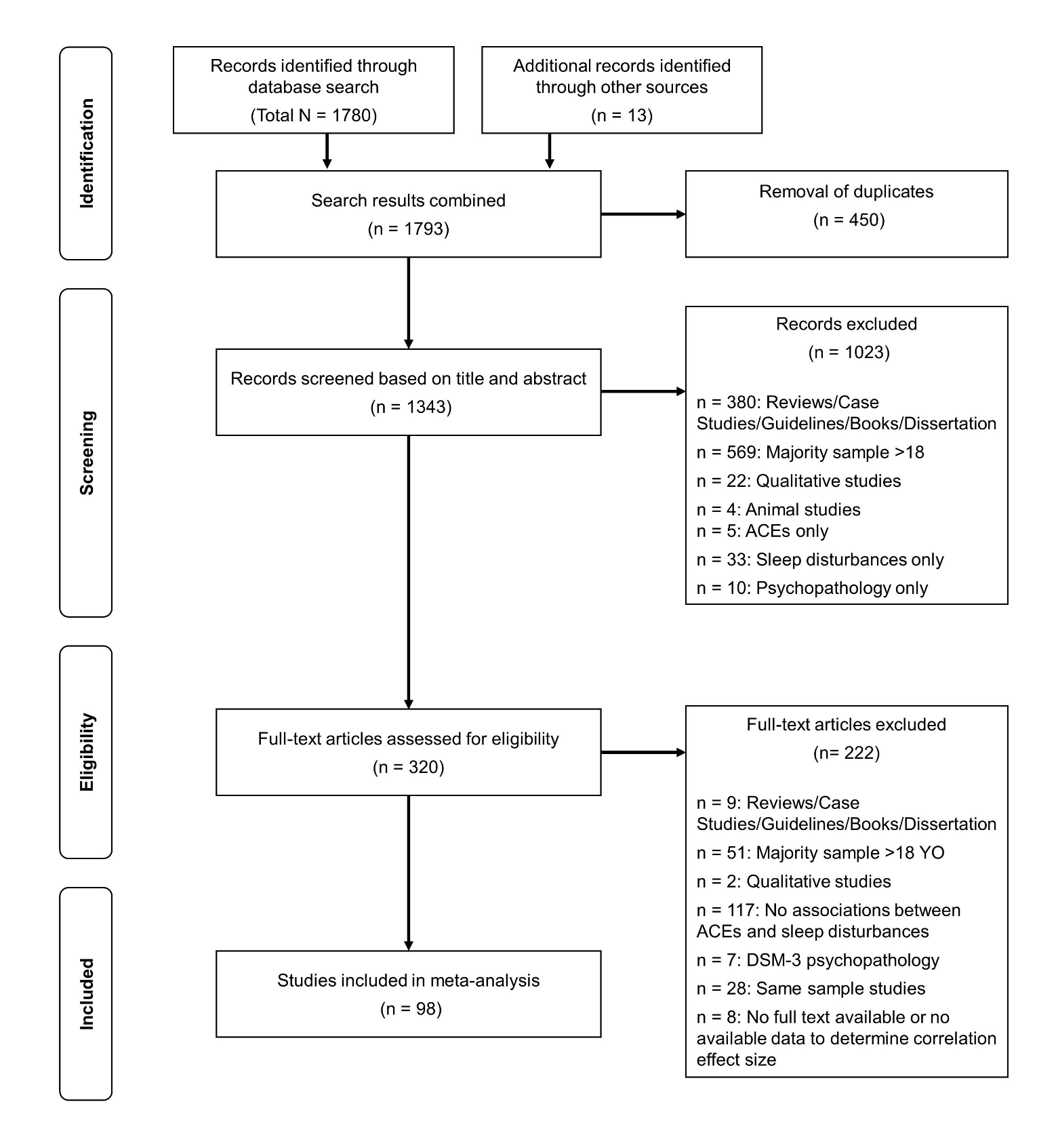
**

Figure S1. Flowchart of Study Selection.


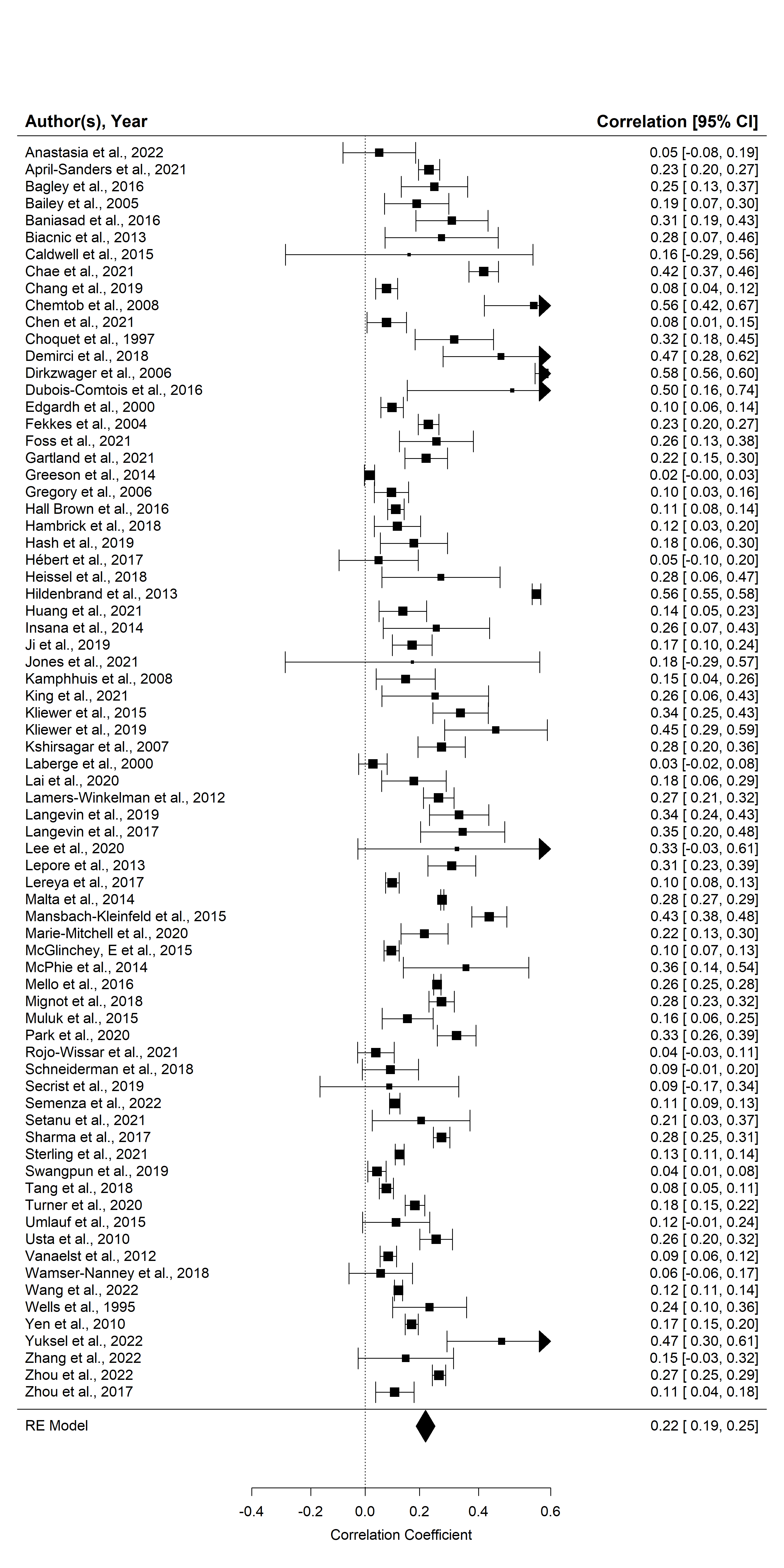


Figure S2. Forest plot for the association between ACEs and sleep disturbance (a path).

‌


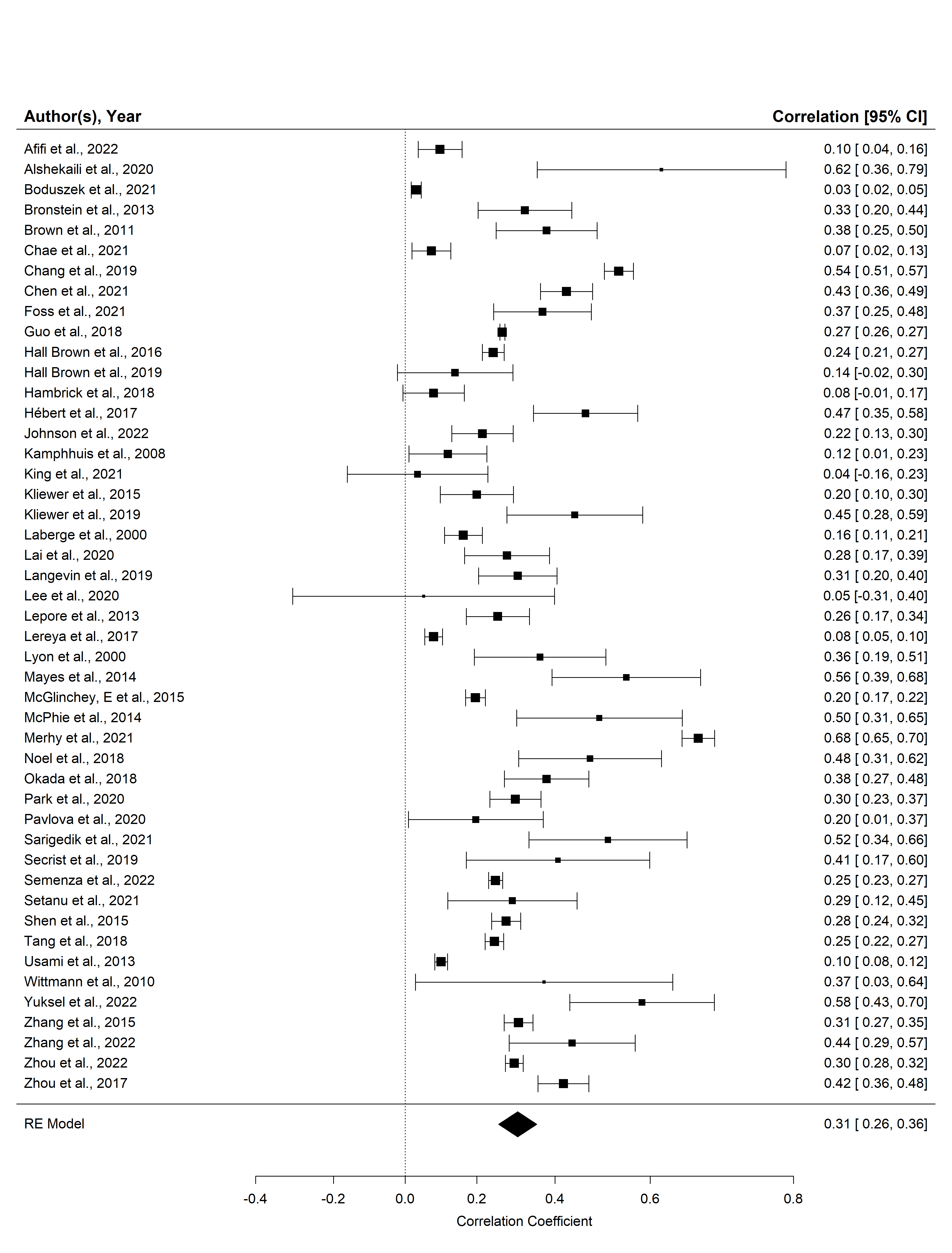


Figure S3. Forest plot for the association between sleep disturbance and psychopathology (b path).


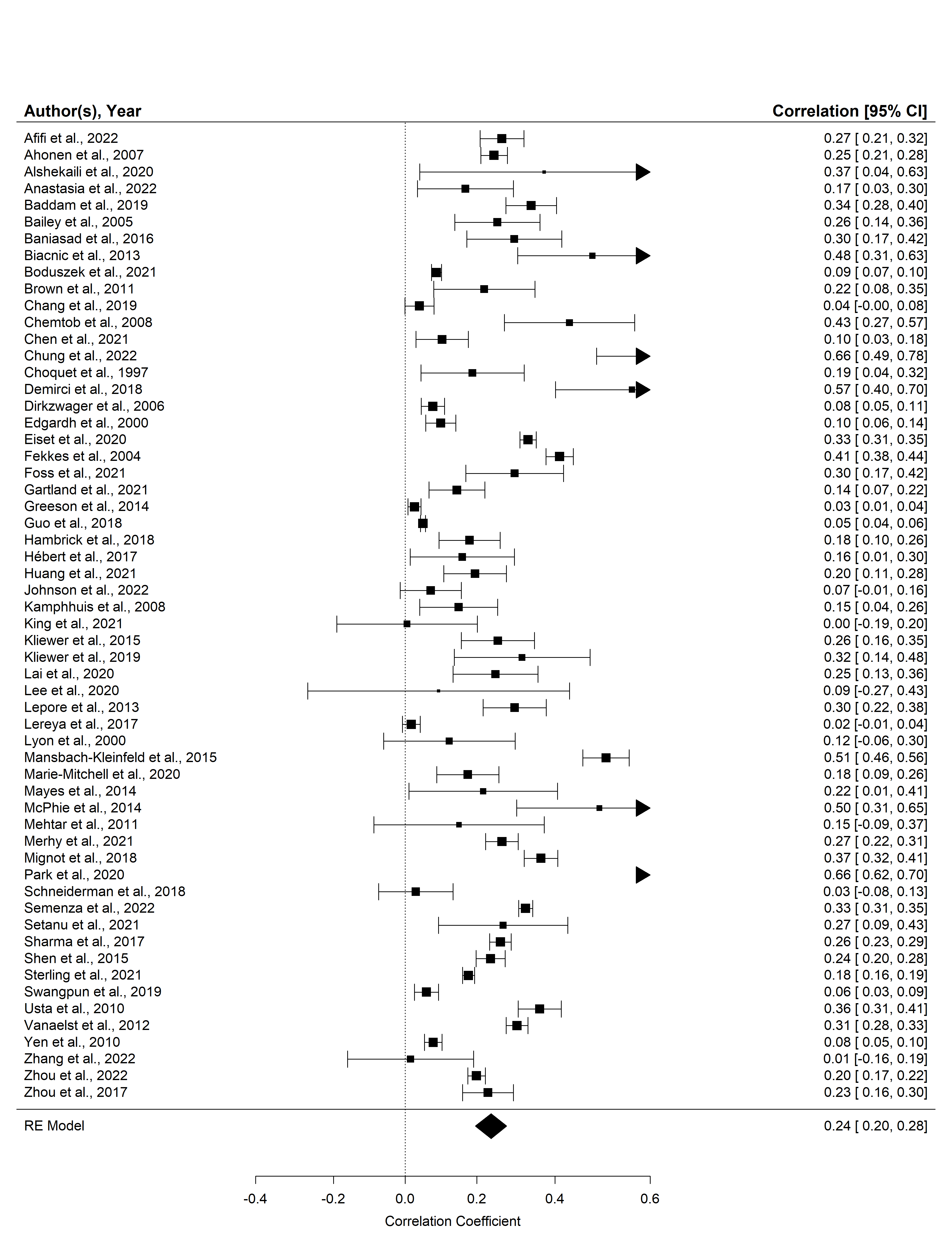


Figure S4. Forest plot for the association between ACEs and psychopathology (c path).
